# Biophysical Characterization of the Leukemic Bone Marrow Vasculature Reveals Benefits of Neoadjuvant Low-Dose Radiation Therapy

**DOI:** 10.1016/j.ijrobp.2020.08.037

**Published:** 2020-08-22

**Authors:** Jamison Brooks, Bijender Kumar, Darren M. Zuro, Jonathan D. Raybuck, Srideshikan Sargur Madabushi, Paresh Vishwasrao, Liliana Echavarria Parra, Marcin Kortylewski, Brian Armstrong, Jerry Froelich, Susanta K. Hui

**Affiliations:** * Department of Radiation Oncology, City of Hope, Duarte, California; † Department of Radiation Oncology, University of Minnesota, Minneapolis, Minnesota; ‡ Hematology Malignancies and Stem Cell Transplantation Institute, City of Hope National Medical Center, Duarte, California; § Regulatory and Quality Solutions, LLC, Monroeville, Pennsylvania; ∥ Department of Immuno-Oncology, City of Hope, Duarte, California; ¶ Beckman Research Institute of City of Hope, Duarte, California; # Department of Development and Stem Cell Biology, City of Hope, Duarte, California; ** Department of Radiology, University of Minnesota, Minneapolis, Minnesota

## Abstract

**Purpose:**

Although vascular alterations in solid tumor malignancies are known to decrease therapeutic delivery, the effects of leukemia-induced bone marrow vasculature (BMV) alterations on therapeutic delivery are not well known. Additionally, functional quantitative measurements of the leukemic BMV during chemotherapy and radiation therapy are limited, largely due to a lack of high-resolution imaging techniques available preclinically. This study develops a murine model using compartmental modeling for quantitative multiphoton microscopy (QMPM) to characterize the malignant BMV before and during treatment.

**Methods and Materials:**

Using QMPM, live time-lapsed images of dextran leakage from the local BMV to the surrounding bone marrow of mice bearing acute lymphoblastic leukemia (ALL) were taken and fit to a 2-compartment model to measure the transfer rate (K_trans_), fractional extracellular extravascular space (V_cc_), and vascular permeability parameters, as well as functional single-vessel characteristics. In response to leukemia-induced BMV alterations, the effects of 2 to 4 Gy low-dose radiation therapy (LDRT) on the BMV, drug delivery, and mouse survival were assessed post-treatment to determine whether neoadjuvant LDRT before chemotherapy improves treatment outcome.

**Results:**

Mice bearing ALL had significantly altered K_trans_, increased V_ec_, and increased permeability compared with healthy mice. Angiogenesis, decreased single-vessel perfusion, and decreased vessel diameter were observed. BMV alterations resulted in disease-dependent reductions in cellular uptake of Hoechst dye. LDRT to mice bearing ALL dilated BMV, increased single-vessel perfusion, and increased daunorubicin uptake by ALL cells. Consequently, LDRT administered to mice before receiving nilotinib significantly increased survival compared with mice receiving LDRT after nilotinib, demonstrating the importance of LDRT conditioning before therapeutic administration.

**Conclusion:**

The developed QMPM enables single-platform assessments of the pharmacokinetics of fluorescent agents and characterization of the BMV. Initial results suggest BMV alterations after neoadjuvant LDRT may contribute to enhanced drug delivery and increased treatment efficacy for ALL. The developed QMPM enables observations of the BMV for use in ALL treatment optimization.

## Introduction

The bone marrow vasculature (BMV) is an important component of the bone marrow to consider for the treatment of hematopoietic malignancies. Malignancy-related vessel remodeling can result in changes in BMV functional characteristics for some hematologic malignancies.^1,2^ As a result, many malignant environments are both hypoxic and inhibit drug delivery, rendering both radiation therapy (RT) and chemotherapy treatments less effective.^3–5^ Combination therapies using a variety of therapeutics to both overcome treatment resistance and enhance tolerability are increasingly used in cancer treatment.^6^ High-dose RT is a possible treatment that has been used for several high-risk hematopoietic malignancies, often as a myeloblative conditioning regimen for hematopoietic stem cell transplantation.^7^ However, RT is not often considered for induction therapy in many hematopoietic malignancies.^8^ For induction therapy, non-myeloblative low-dose RT (LDRT) is a potential option for combination with chemotherapy and has several attractive benefits, including the radiosensitivity of hematological malignancies, the ability to precisely target regions of high disease burden, and lower toxicity compared with myeloblative high-dose RT.^9,10^ Such advantages make LDRT a possible candidate for combination therapy; however, the response of the BMV to LDRT is largely unknown and needs to be considered to optimize the delivery of therapeutics to the malignant disease of the bone marrow.

The optimization of therapeutic delivery is challenging because of the heterogeneity of malignant vasculature and the complex response of the vasculature to treatment.^11^ To effectively optimize therapeutic delivery, both the pharmacokinetics of the therapeutics and the functional vascular characteristics (eg, permeability, density, morphology, and perfusion) must be understood. Attempts to optimize the BMV on the basis of pharmacokinetics alone may result in further vascular dysfunction.^12^ Alternatively, optimization based on solely the BMV characteristics does not account for the wide variety of sizes and types of therapeutics (target or nontargeted).^13^ Therefore, effective methods that simultaneously observe vascular functional characteristics and therapeutic pharmacokinetics are essential to condition the vasculature in combination therapy.

Although methods to assess therapeutic pharmacokinetics (eg, dynamic fluorescence imaging, dynamic contrast-enhanced imaging) exist,^14,15^ limitations are involved. First, such methods do not have the resolution required to identify the features of capillary-sized vessels, making observations of the underlying vascular functional characteristics difficult or impossible.^16^ Second, the resolution of such modalities often makes it challenging to observe tracer uptake in the bone marrow of a mouse model, limiting usefulness in a preclinical setting for hematologic applications. Alternatively, microscopic imaging modalities can identify the vascular functional characteristics but have not been used thoroughly to quantitatively assess therapeutic pharmacokinetics using kinetic compartmental modeling.

To address the need for a single imaging platform capable of observing the BMV functional characteristics and pharmacokinetic alterations from LDRT, we developed and validated compartmental modeling for quantitative multiphoton microscopy (QMPM) time-lapsed imaging of the BMV in the calvarium of live mice bearing GFP+ Philadelphia chromosome B-cell acute lymphoblastic leukemia (ALL). The calvarium region was selected for QMPM because (1) it contains sinusoidal vessels similar to those in other hematopoietic bone marrow regions^17,18^; (2) it has similar proportions of hematopoietic stem cells and similar hematopoietic reconstitution potential compared with bone marrow regions that are considered the primary sites of hematopoiesis^19^; and (3) it is a flat superficial bone requiring minimal invasive surgery before live imaging. For QMPM, time-lapsed images of fluorescent dextran leakage from the vascular lumen to the interstitial tissue were fit to a compartmental kinetic model to quantitatively assess (1) the kinetic transfer rate of dextran to the tissue space (K_trans_), (2) the fractional extracellular extravascular space (V_cc_), and (3) vascular permeability, while also monitoring functional BMV characteristics including single-vessel blood perfusion, functional vessel surface area, vascular morphology, and vascular density.

After seeing changes in the BMV with the onset of ALL, we hypothesized that LDRT (2–4 Gy) may improve vascular function and morphology and reduce leukemic burden, leading to better uptake of therapeutics and enhanced efficacy when used as a neoadjuvant therapy before therapeutic treatment. The response of the BMV to LDRT in mice bearing ALL was assessed using 2 treatment types: (1) targeted LDRT to half of the calvarium and to the left femur (tLDRT) and (2) total body irradiation (TBI). tLDRT was used to only irradiate small volumes of interest, ensuring that the systemic blood perfusion of the cardiovascular system was not affected with treatment.

To assess the uptake of therapeutics, cellular uptake of fluorescent agents in regions receiving tLDRT and matching regions outside the radiation field (abscopal) were compared to assess therapeutic delivery. Assessments were made 2 days after tLDRT and 20 minutes after intravenous injection of Hoechst 33342 dye (Hoechst, MW: 615.99) and daunorubicin HCL (daunorubicin, MW: 563.98), a chemotherapy regimen used in the treatment of ALL and other hematologic malignancies.^20^ Both agents are of similar size to many small-sized therapeutic agents used for the treatment of hematologic malignancies.^8^ In addition, the efficacy of nilotinib (MW: 529.52), a similar-sized therapeutic agent effective for the treatment of ALL,^20^ was administered before or after 4 Gy TBI to determine whether LDRT-related changes to the malignant bone marrow and BMV may have an impact on treatment outcome in combination therapy.

## Methods and Materials

### Mice, cell lines, and drugs

All procedures for animal experimentation were performed according to City of Hope guidelines and approved by the Institutional Animal Care and Use Committee. C57BL/6J mice (strain 000664, Jackson Laboratory, Bar Harbor, ME) mice were injected with 1 × 10^6^ GFP+BCR-ABL (p190Kd) expressing B-cell ALL cells,^21^ suspended in 200 μL of phosphate-buffered saline (PBS), through the tail vein. To test drug delivery, injection concentrations of 10 mg/kg and 40 mg/kg were used for fluorescent Hoechst 33342 dye (Thermo Fisher Scientific, Waltham, MA) and fluorescent daunorubicin HCL (Selleck Chemicals LLC Houston, TX), respectively. Mice were imaged or euthanized for tissue processing 20 minutes after tail vein injection of Hoechst or daunorubicin. Nilotinib (LC Laboratories, Woburn, MA) treatments for survival studies were given at 50 mg/kg using oral gavage. Further details can be found in the Appendix1A.

### Mouse surgery, multiphoton imaging, and image processing

For QMPM calvarium imaging, mice were anesthetized using isoflurane, and surgery was performed to remove the skin and periosteum above the cranium. A custom titanium head plate was affixed directly to the calvarium and inserted directly into a heated microscope stage for stability during imaging.^22^ Imaging was performed with a Prairie Ultima multiphoton microscope (Bruker Corporation, Billica, MA). All imaging was performed at single-day time points, and mice were euthanized after imaging sessions. Vascular blood pool imaging was performed with intravenous injections of 20 μL of Qtracker 655 Vascular Label (Invitrogen, Carlsbad, CA) added to 80 to 180 μL of phosphate-buffered saline. Second harmonic generation imaging was used to observe the collagen in the bone regions and identify pockets of calvarium marrow within the bone. Velocities of cells flowing through the BMV network were measured using QMPM and calculated in a similar fashion as in previous works.^18,23^ Static image processing and tiling of large image sets were performed using Fiji/ImageJ^24,25^ and CellProfiler (v.2.2.0).^26^

Vascular permeability, K_trans_, and V_ec_ were measured by time-lapsed imaging of 150-kDa TRITC dextran (TdB Consultancy, Uppsala, Sweden) leakage through the BMV. Mice were intravenously catheterized and injected with 500 μg of TRITC dextran during image acquisition. To identify blood and tissue compartments and obtain time-curves of dextran fluorescent intensity, a series of automatic thresholds was calculated for time-lapsed images using the Otsu method^27^ (Fig. E1A and E1B) in Fiji/image J. Spatial image drift over time was accounted for by coregistration of image frames using Matlab (R2018a 9.41.0.81364, MathWorks Natick, MA). A 2-compartment model with fixed equal transport rates in and out of the tissue compartment was used to model dextran time-curves from blood and tissue compartments.^28^ Compartmental modeling of dextran time-curves was performed in Matlab to obtain K_trans_ and V_ec_. Vascular permeability was determined by dividing K_trans_ by the vascular surface area per image volume. A detailed description of mouse surgery, image acquisition, and image analysis can be found in Appendix 1B-D.

### Blood pressure measurements

Pulse rate, systolic blood pressure, and diastolic blood pressure measurements were acquired by tail cuff method on conscious mice using the BP-2000 Blood Pressure Analysis System (Vistech Systems). See Appendix 1E for details.

### Computed tomography and LDRT treatments

Computed tomography (CT) images were administered and acquired with the Precision X-RAD SMART Plus/225cx small animal image-guided irradiation system (Precision X-Ray, North Branford, CT).^29^ CT image processing, contouring, and visualization were performed with Vivoquant (InviCro, Boston, MA) software. Dose calculations were performed with the small animal RT planning system^29^ using a Monte Carlo dose engine.^30,31^

Mice were administered tLDRT treatments in a single fraction of 2 to 4 Gy soft tissue–equivalent dose to the left half of the calvarium and left femur. tLDRT treatments were used to assess drug delivery and disease burden by comparing treated regions (left femur and left half of the calvarium) and abscopal regions (right femur and right half of the calvarium). TBI single-fraction treatments of 2 to 4 Gy were administered at matching soft tissue doses to tLDRT treatments, enabling comparisons of the irradiated BMV in the left half of the calvarium for tLDRT treatments (local vascular treatment) and TBI treatments (systemic cardiovascular treatment). All imaging and tissue harvests were performed 40 to 48 hours postirradiation. See Appendix 1F for more details.

### Flow cytometry

Endothelial cell analysis was performed according to Kumar et al.^32^ Briefly, endothelial cells were quantified as CD45/TER119− CD31+ identified from crushed femur and tibia bone samples. Bones were incubated in collagenase to liberate the leukemic and bone marrow microenvironment cells from the bone surface. Measurements of endothelial cell counts, cellular uptake of Hoechst, cellular uptake of daunorubicin, and GFP+ ALL cell counts were performed in standard fashion. Complete details can be found in Appendix 1G.

### Dextran well plate readings

Well plate fluorescence readings of TRITC dextran concentration were read from supernatants taken from crushed single-femur cell suspensions after removal of cells with centrifugation. Further details are found in Appendix 1H.

### Statistical analysis

All statistical testing was performed using Prism (V.7.01, GraphPad). Unless otherwise noted, significance testing between groups was performed using the Welch 2-sided *t* test with a *P* value < .05 considered significant. Significance testing for survival data was calculated using the log-rank Mantel-Cox test. For linear correlation testing, the R^2^ coefficient of determination tests were used. All distribution error bars are displayed as the mean ± 1 standard error of the mean.

## Results

### Bone marrow microenvironment remodeling and leukemia localization during ALL growth

**Fig. 1.**
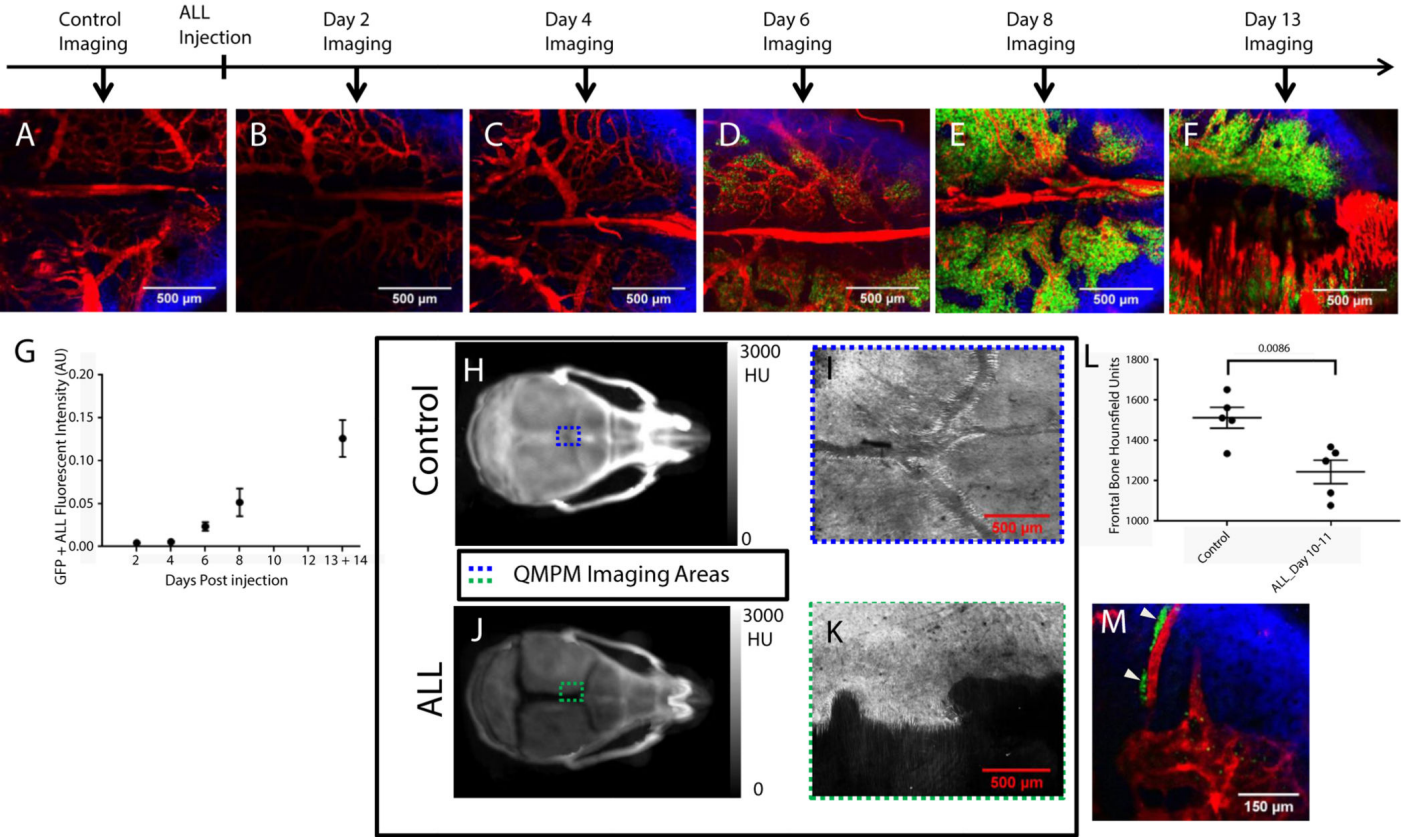
Multiphoton microscopy imaging of acute lymphoblastic leukemia (ALL) progression and bone degradation in mouse calvarium. Tiled multiphoton microscopy images of the calvarium in (A) a healthy control mouse and mice 2, 4, 6, 8, and 13 days after BCR-ABL injection are shown (B-F). Red is Qtracker 655-labeled vascular blood pool fluorescence, green is green fluorescent protein (GFP) ALL fluorescence, and blue is the second harmonic generation image of the collagen in the bone. (G) GFP+ALL fluorescent intensity in images from mouse calvarium, their respective days postinjection (1 time point per mouse, n = 4, 4, 4, 3, and 3 mice for each time point, respectively). (H, J) Three-dimensional intensity projection images of computed tomography scans from healthy control mice and mice bearing ALL 12 days post-ALL injection. Colored dotted lines indicate relative regions of multiphoton microscopy imaging performed in I and K. (I, K) Multiphoton microscopy images of the second harmonic generation of the collagen in the bone are shown from a healthy control mouse and a mouse bearing ALL 14 days post-ALL injection. (L) Computed tomography Hounsfield units from contoured frontal bones of healthy control mice and mice bearing ALL 10 to 11 days post-ALL injection (n Z 5 mice per group). (M) A region of heterogeneous ALL distribution. Pockets of heterogeneous ALL are marked with white triangles.

Large QMPM 3-dimensional tiled images of separate mice showed steady progression of ALL disease burden in the calvarium from 2 to 14 days post-ALL injection (Fig. 1A-G; Video E1). Degradation of the calvarium was present in mice with a high ALL burden (Fig. 1H and 1J), resulting in a loss of second harmonic generation signal from the collagen in the bone near the sagittal and coronal calvarium sutures (Fig.1I and 1K). Additionally, x-ray CT scans revealed reduced Hounsfield units on the frontal lobe of mice bearing ALL compared with healthy control mice, suggesting a loss of bone mineral density with the onset of ALL (Fig. 1L). ALL was predominately homogenously distributed throughout calvarium marrow space. However, occasional regions of the calvarium did contain heterogeneous pockets of ALL (Fig. 1M).

### Changes in K_trans_ and increased BMV permeability in mice bearing ALL

**Fig. 2.**
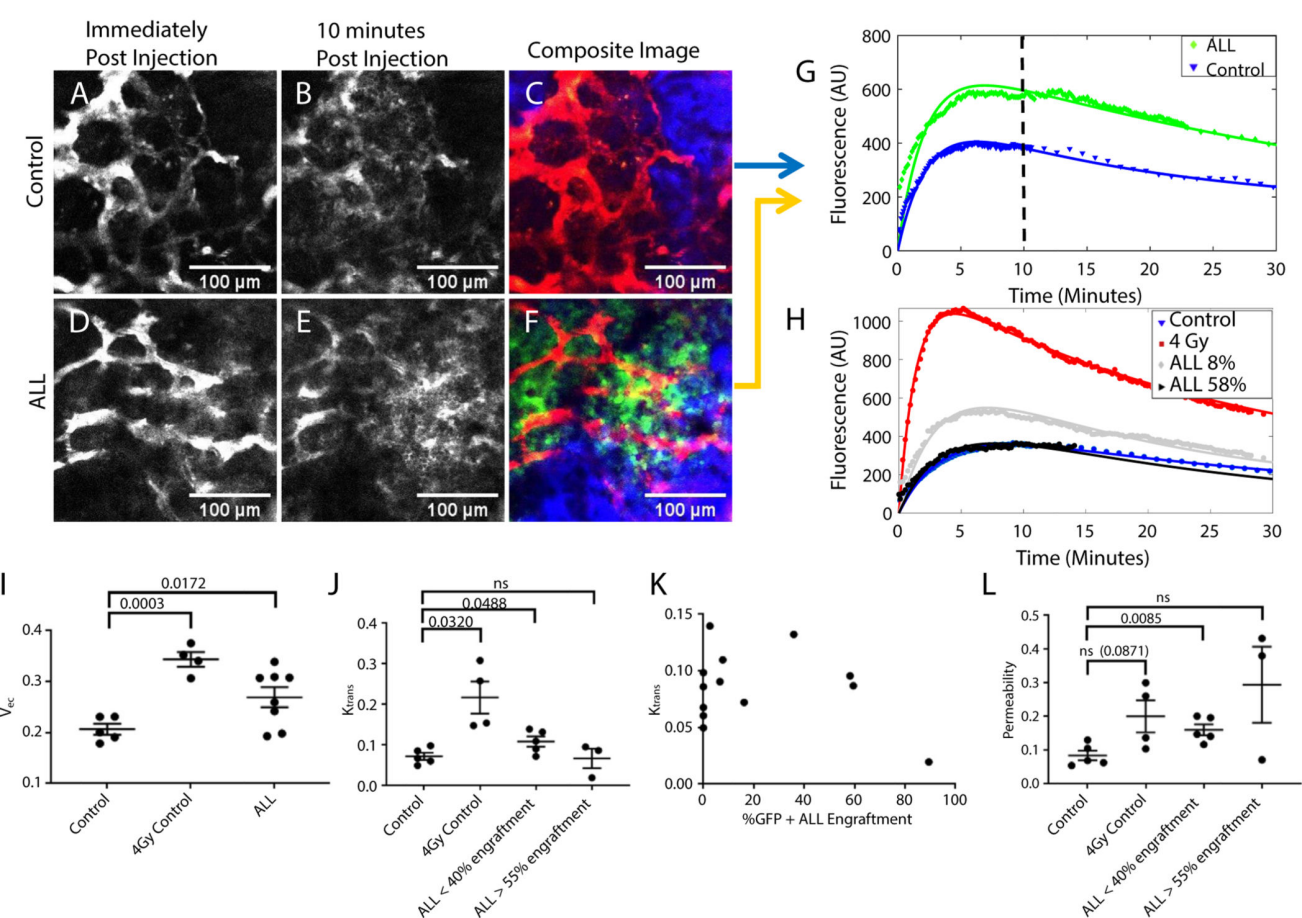
Quantitative multiphoton microscopy imaging measures K_trans_, V_ec_, and vascular permeability. (A, B, D, E) TritC dextran fluorescence is shown for a healthy mouse and a mouse bearing acute lymphoblastic leukemia (ALL) immediately and 10 minutes after dextran injection. (C, F) Merged images verifying the local presence of ALL are shown. Green is green fluorescent protein (GFP)+ ALL fluorescence, red is TritC dextran fluorescence, and blue is the second harmonic generation image of the collagen in the bone. (G) Time-lapsed dextran fluorescent intensity from segmented tissue compartment regions is plotted with corresponding fitted tissue compartment curves for the control mouse and mouse bearing ALL shown in A-F. The vertical striped line indicates the 10 minutes postinjection time point depicted in the images. (H) A plot of time-lapsed dextran fluorescent intensities from the segmented tissue compartments of a healthy control mouse, a nonleukemic 4 Gy targeted low-dose radiation therapy (tLDRT)-treated mouse, a mouse with 8% ALL femoral bone marrow engraftment, and a mouse with 58% ALL femoral bone marrow engraftment. The fitted tissue compartment curves for each mouse are also plotted. Matching time-lapsed movies and matching normalized time-lapsed data are displayed in Videos E2-4 and Figure E1D. (I) Values for V_ec_ are shown for healthy control mice, nonleukemic LDRT-treated mice, and mice bearing ALL (n = 5, 4, and 8 mice per group, respectively). (J, L) Values for K_trans_ and vascular permeability are shown for healthy control mice, nonleukemic tLDRT-treated mice, mice with less than 40% ALL femoral bone marrow engraftment, and mice with more than 55% ALL femoral bone marrow engraftment. (n = 5, 4, 5, and 3 mice, respectively). (K) A plot of ALL femur bone marrow engraftment compared with K_trans_ for healthy control mice and mice bearing ALL is shown (n = 13 mice).

For all time-lapsed QMPM images, dextran had reached the BMV between 6 and 12 seconds after injection. The fluorescent intensity of dextran in time-lapsed images was well described by biexponential decay (Fig. E1C), suggesting semiconsistent BMV perfusion. Dextran uptake into the extravascular calvarium bone marrow tissue started immediately after dextran perfusion into the BMV (Fig. 2A-H, Fig. E1C and E1D, Videos E2-4). The time-lapsed fluorescent intensity of dextran in blood and tissue compartments showed good agreement with the compartmental model used (Fig. E1E). Validation of the compartmental model was performed by perturbing the system using tLDRT to the left femur and left half of the calvarium in healthy mice, as radiation has been shown to both increase V_ec_ (by decreasing total tissue cell number) and damage vasculature, causing increased leakage.^33–35^ A dose of 4 Gy tLDRT significantly increased V_ec_ and K_trans_ in tLDRT-treated calvarium regions compared with regions in untreated healthy mice (*P* = .0003 and *P* = .0320, respectively, Fig. 2I-J). A corresponding reduction in total cell number in irradiated femur and calvarium bone marrow regions was found compared with abscopal regions (Fig. E2A and E2B). Significantly higher dextran fluorescence in crushed femur supernatants was found for tLDRT-treated femurs compared with abscopal femurs (*P* = .0003, Fig. E2C), suggesting LDRT significantly increases total dextran concentration in the bone marrow tissue. A negative correlation between femoral bone marrow total cell count and dextran fluorescence of crushed femur supernatants was found, confirming that extracellular space and the total dextran uptake in the bone marrow are influenced by total cellular density (R^2^ = 0.7905, *P* < .0001, Fig. E2D).

After validation, time-lapsed QMPM images in the calvarium of mice bearing ALL revealed an increase in V_ec_ compared with healthy control mice (*P* = .0172, Fig. 2I). No significant difference in femoral bone marrow total cell number was observed (*P* = .1744), indicating that the change in V_ec_ may be due to other additional factors, such as bone degradation or high interstitial fluid pressure. At low-to-moderate ALL engraftment, K_trans_ was significantly higher than in healthy control mice (*P* = .0488) (Fig. 2J and 2K). Functional BMV surface area per volume in time-lapsed QMPM images decreased with ALL burden (Fig. E2E). A significant increase in vascular permeability was observed in mice with low-to-moderate ALL engraftment compared with healthy control mice (*P* = .0085, Fig. 2L, Fig. E2F).

Time-lapsed QMPM imaging of daunorubicin was performed in a mouse bearing ALL and compared with dextran kinetics to ensure that QMPM-based, time-lapsed imaging can be used for fluorescent agents of varying sizes and pharmacokinetic uptake patterns. A clear distinction between vascular, tissue, and cellular compartments could be seen in daunorubicin images (Fig. E3A-J, Video E5). Faster blood clearance and faster peak tissue uptake of daunorubicin is observed compared with dextran (because of its smaller molecular weight) (Fig. E3K).

### BMV remodeling results in angiogenesis, poor perfusion, and loss of functional vessels with ALL

**Fig. 3.**
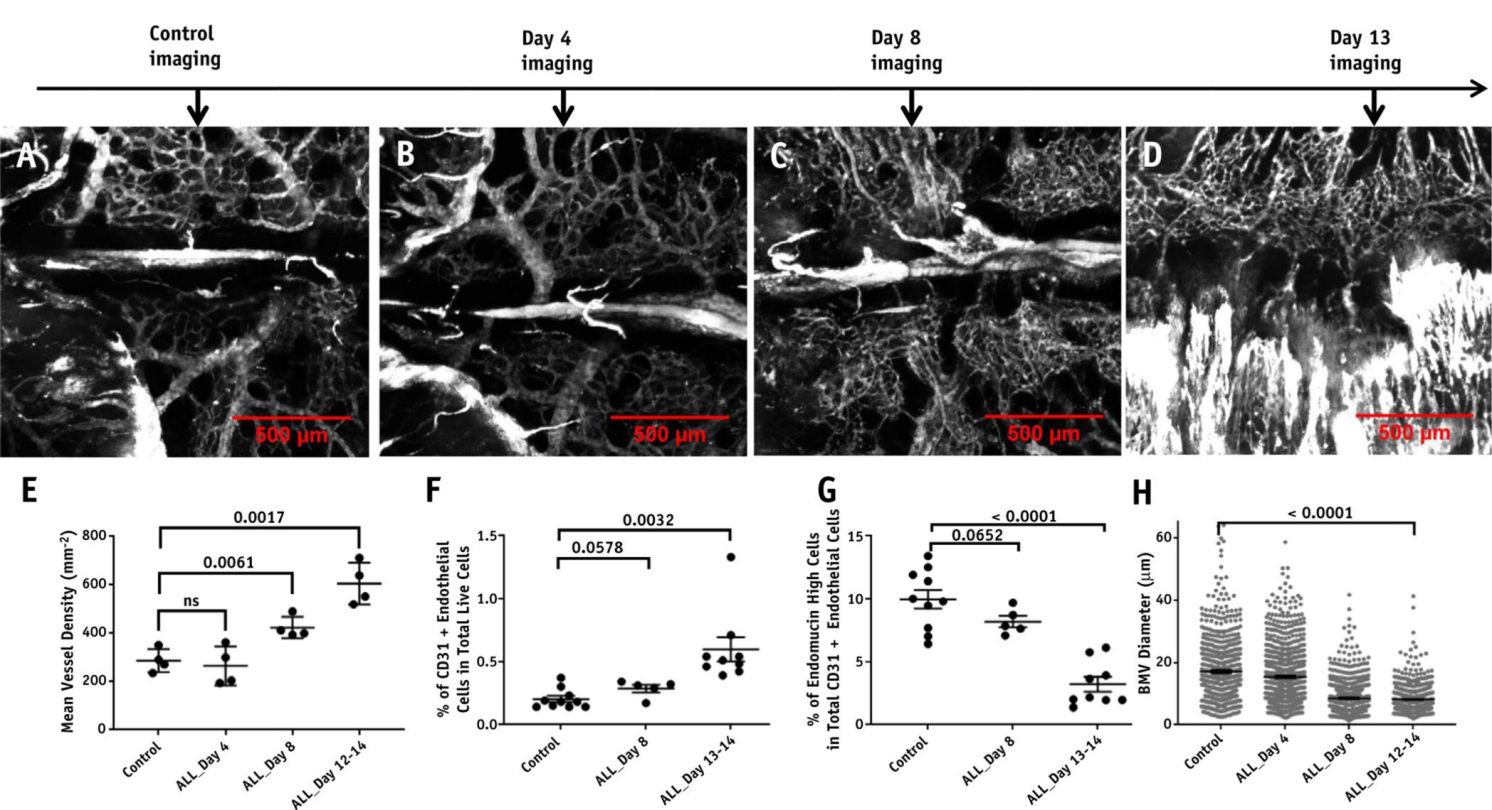
Bone marrow vasculature measurements of vessel morphology with the onset of ALL. Representative tiled maximum intensity projection images of Qtracker 655 vascular blood pool fluorescence for (A) control mice and mice (B) 4 days, (C) 8 days, and (D) 13 days after acute lymphoblastic leukemia (ALL) injection are shown. (E) Mean vessel density measurements from images of healthy control mice and BCR-ABL mice 4, 8, and 12 to 14 days post-ALL injection are shown (n = 4 mice per group). (F) Flow cytometry analysis of the percentage of CD31+ endothelial cells in total live cells for control, 8 days, and 13 to 14 days post-ALL injection are shown (n = 10, 5, and 9 mice per group, respectively). (G) The fraction of high endomucin-expressing endothelial cells in total endothelial cells for control, 8 days post-ALL injection, and 13 to 14 days post-ALL injection mice is shown (n = 10, 5, and 9 mice per group, respectively). (H) Measurements of vessel diameter from images of healthy control and ALL burdened mice 4, 8, and 12 to 14 days post-ALL injection are shown (n = 4 mice per group).

QMPM measurements of calvarium mean vessel density were higher in mice bearing ALL compared with those in healthy control mice (319 mm^−2^, *P* = .0017, Fig. 3A-E). Crushed femur and tibia samples were analyzed with flow cytometry to observe endothelial cell changes with ALL progression. An increase in the CD45/TER119− CD31+ endothelial cell fraction in total live cells was observed in mice bearing ALL compared with control mice (*P* = .0032, Fig. 3F, Fig. E4A). No significant differences in femoral bone marrow total cell number between healthy mice and mice bearing ALL were observed, suggesting an overall increase in the total number of endothelial cells present in the bone marrow of mice bearing ALL (*P* = .1744, Fig. E4B). Additionally, a significant reduction in the high endomucin expressing endothelial cell fraction in total endothelial cells was found for mice bearing ALL compared with control mice (*P* < .0001, Fig. 3G; Fig. E4C). Endothelial cells with high endomucin expression have previously been reported to colocalize with osteoblast progenitors in the growth plate regions of the femoral bone and are responsible for hematopoietic stem cell (HSC) maintenance by secreting HSC-supporting cytokines.^36^ These data suggest that ALL transforms the endothelial subset away from a hematopoietic supporting niche in support of ALL.

Endothelial expansion was accompanied by large changes in BMV function and morphology. QMPM revealed a significant reduction (8.93 μm) in the average inner diameter of vessels in the calvarium in mice bearing ALL compared with healthy control mice (*P* < .0001, Fig. 3H). Average velocities of cells flowing through the BMV of mice bearing ALL (1.34 ± 0.11 mm/s) were reduced compared with healthy control mice (3.70 ± 0.484 mm/s, *P* < .0001). Control mice typically had small inner diameter vessels with rapid blood flow connected to slow flowing vessels with a large inner diameter. Alternatively, mice with moderate-to-high ALL burden displayed a more homogenous population of vessels in terms of both blood flow velocity and vessel diameter, suggesting that typical cellular extravasation, oxygen delivery, and drug delivery may be altered in mice bearing ALL (Fig. E5A-H).

QMPM imaging revealed regions of semicollapsed vessels in the calvarium of mice as early as day 8 after ALL injection (Fig. E6A and E6B). Accumulation of nonmoving cells in blood vessels was observed near regions of partial BMV collapse (Fig. E6C). The BMV opened and closed intermittently in some mice bearing ALL (Video E6), but not in control mice (Video E2). Measurements of systolic and diastolic pressure were taken to better understand the mechanics behind BMV closing. Reductions in systolic (56.2 mm Hg) and diastolic pressure (31.0 mm Hg) were measured with the onset of ALL (*P* = .0002 and 0.0375, respectively, Fig. E7A-D), suggesting that reduced systemic blood pressure may contribute to BMV collapse.

### LDRT improves blood perfusion, improves chemotherapy delivery, and increases survival in combination therapy

**Fig. 4.**
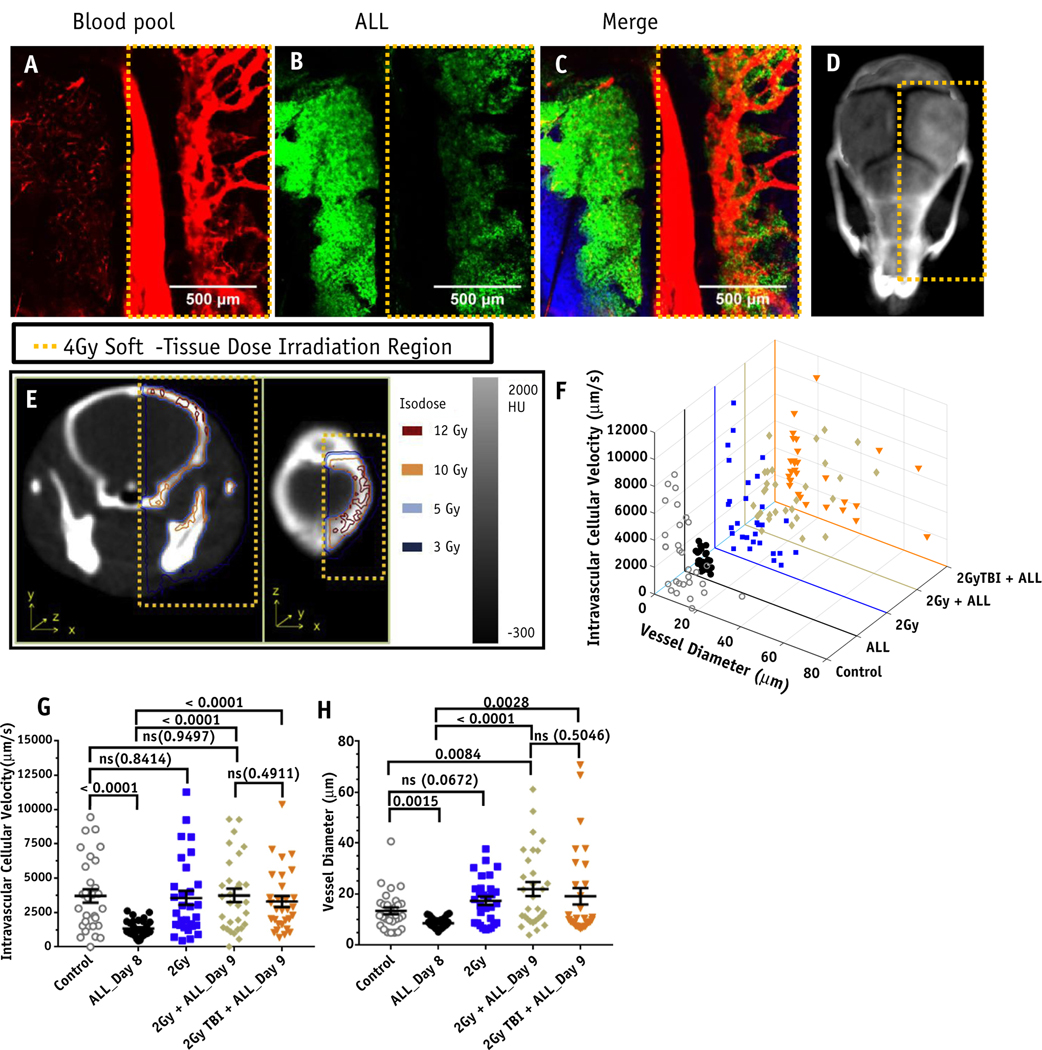
Bone marrow vasculature perfusion and morphology measurements in mice bearing acute lymphoblastic leukemia (ALL) after low-dose radiation therapy (LDRT) treatments. Tiled images taken 2 days after 4 Gy targeted LDRT (tLDRT) and 13 days post-ALL injection, showing irradiated and abscopal regions of the mouse calvarium with (A) Qtracker 655 vascular blood pool fluorescence in red, (B) green fluorescent protein (GFP)+ ALL fluorescence in green, and (C) a merged image with the second harmonic generation image of the collagen in the bone in blue. (D) A representative 3-dimensional intensity projection CT image depicting the tLDRT treatment region. (E) Computed tomography images with isodose lines showing the corresponding 4 Gy tLDRT irradiated calvarium region shown in A-C. (F-H) Intravascular cellular velocity and vessel diameter plots for individual blood vessels are plotted for healthy control mice, nontreated mice bearing ALL, 2 Gy tLDRT-treated healthy mice, 2 Gy tLDRT-treated mice bearing ALL, and 2 Gy TBI-treated mice bearing ALL (n = 3 mice per group). Ten individual vessel measurements were taken from each mouse. Untreated and irradiated mice bearing ALL were imaged 8 days and 9 days post-ALL injection, respectively.

Next, the effects of LDRT on the BMV in mice bearing ALL were tested with the hypothesis that LDRT would be beneficial in (1) improving the function and morphology of the BMV, (2) enhancing therapeutic uptake, and (3) increasing treatment efficacy when used before the administration of therapeutics. tLDRT treatments altered the morphology and functionality of the BMV in mice bearing ALL 2 days post-treatment (Fig. 4A-E). Mice bearing ALL and treated with 2 Gy tLDRT had increased average vascular diameter (13.4 μm) and increases in the average velocity of cells flowing through the BMV network (2.41 mm/s) compared with untreated mice bearing ALL (*P* < .0001 and 0.0001, respectively, Fig. 4F-H). These treatments increased average cellular vascular velocity to levels similar to those in healthy control mice (*P* = .9497). Similar BMV changes were observed with 2 Gy TBI treatments, suggesting that systemic LDRT to the cardiovascular system does not largely affect blood flow and that functional vascular changes may be beneficial for a variety of systemic or targeted RT treatments.

**Fig. 5.**
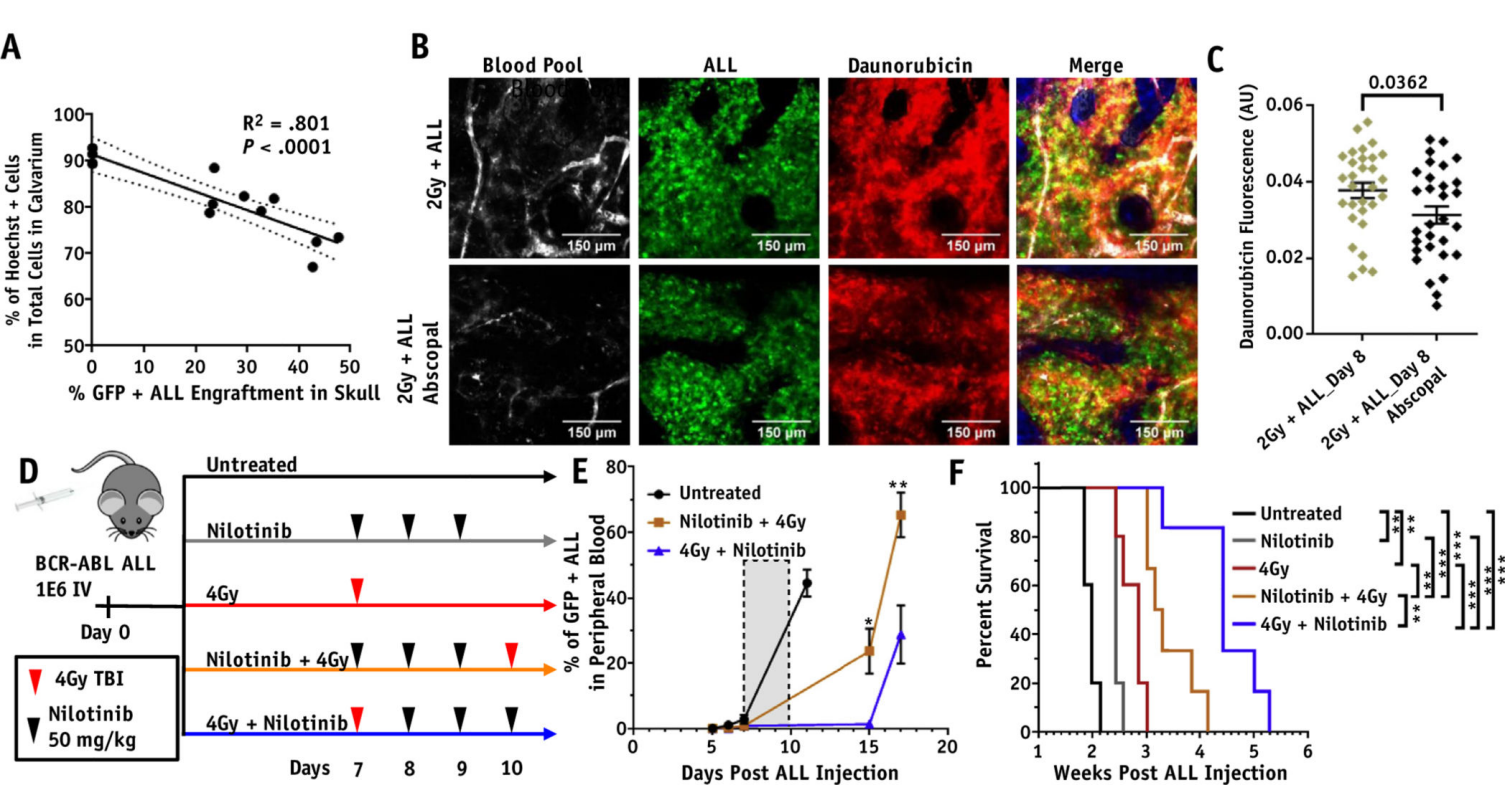
Therapeutic uptake after neoadjuvant low-dose radiation therapy (LDRT) and treatment efficacy of combination therapy. (A) Flow cytometry analysis of the percentage of total green fluorescent protein (GFP)+ acute lymphoblastic leukemia (ALL) cells stained positive for Hoechst in abscopal calvarium regions are plotted (n = 13 mice). The corresponding R^2^ value and nonzero significance value are shown. (B) Multiphoton microscopy images of treated and abscopal calvarium regions for a mouse receiving 2 Gy targeted LDRT (tLDRT). Qtracker 655 vascular blood pool fluorescence is in gray, GFP ALL fluorescence is in green, daunorubicin fluorescence is in red, and the second harmonic generation image of the collagen in the bone displayed in the merged image is in blue. Daunorubicin was injected 20 minutes before imaging. (C) Cellular daunorubicin fluorescence taken from image segmentation of 30 single ALL cells with the highest GFP fluorescent intensity for 2 Gy tLDRT-treated and abscopal regions shown in B. (D) A treatment schema for mice bearing ALL is shown for untreated, nilotinib, 4 Gy total body irradiation (TBI), nilotinib before 4 Gy TBI, and 4 Gy TBI before nilotinib treatment groups. Treatment was started at day 7, when ALL could be detected in the peripheral blood of all the mice. (E) A plot of the percentage of ALL in peripheral blood for untreated (n = 4, 5, 5, and 5 mice per time point for days 5, 6, 7, and 11 respectively), 4 Gy TBI after nilotinib (n = 6 mice per time point), and 4 Gy TBI before nilotinib (n = 6, 6, 6, 4, and 6 mice per time point for days 5, 6, 7, 15, 17, respectively) treatment groups (*P <.05, **P <.01). The gray shaded box indicates days 7 to 10 when treatment was administered. (F) The survival plot for mice bearing ALL receiving no treatment, nilotinib only, 4 Gy TBI only, 4 Gy TBI after nilotinib, and 4 Gy TBI before nilotinib is shown (n = 5, 5, 5, 6, and 6 mice per group, respectively). Significant differences in survival are seen between all treatments except nilotinib only and 4 Gy TBI-only treatments (*P < .05, **P < .01, ***P < .001, ****P < .0001).

After seeing changes in the BMV with tLDRT and TBI, the therapeutic delivery of Hoechst and daunorubicin to the bone marrow was measured by cellular uptake using flow cytometry. Mice were administered intravenous injections of Hoechst or daunorubicin 2 days after 2 Gy and 4 Gy tLDRT and20 minutes before being euthanized for tissue harvest. To observe changes in drug uptake, tLDRT-treated calvarium and femur regions were compared with matching abscopal regions in mice bearing ALL. The percentage of total cells that stained positive for Hoechst in abscopal calvarium regions had a negative correlation with ALL engraftment (Fig. 5A), whereas no significant changes in overall total cell number were seen in the femoral bone marrow of mice bearing ALL compared with healthy mice (*P* = .1744). This suggests that cellular Hoechst uptake is inhibited by the onset of ALL but not reduced due to a change in total cell number. In 2 Gy tLDRT-treated regions compared with abscopal regions, increases in the percentage of ALL cells labeled positive for Hoechst (88.3% ± 3.6% and 63.5% ± 3.0% respectively, *P* = .0067) and daunorubicin (90.8% ± 2.3% and 72.3% ± 1.8% respectively, *P* = .0036) were found (Table 1, Fig. E8A-E). Similar trends were observed with 4 Gy tLDRT treatments. Nonleukemic tLDRT-treated mice showed high percentages of total calvarium marrow cells labeled positive for Hoechst in both 2 Gy tLDRT-treated (94.7% ± 3.9%) and abscopal calvarium regions (90.5% ± 1.0%, Fig. E8F). QMPM-based image segmentation of ALL cells revealed higher daunorubicin fluorescence for cells in 2 Gy tLDRT-treated calvarium compared with abscopal calvarium (*P* = .0362, Fig. 5B and 5C). Results demonstrate enhanced cellular uptake of small-molecular-weight therapeutics after neoadjuvant LDRT.

To test the treatment efficacy of the therapeutic delivery enhancements after neoadjuvant LDRT, we treated mice with 4 Gy TBI in combination with nilotinib. TBI was chosen for treatment because it had similar effects on the BMV as tLDRT and it was more suitable as a treatment regimen. A 4 Gy dose was chosen because it exhibits increased cell killing effects compared with 2 Gy with similar enhancements in therapeutic delivery. A treatment schema is shown in Figure 5D showing untreated, nilotinib, 4 Gy TBI, 4 Gy TBI after nilotinib, and 4 Gy TBI before nilotinib groups. Significantly increased percentages of ALL in the peripheral blood were found in mice treated with 4 Gy TBI after nilotinib compared with mice treated with 4 Gy TBI before nilotinib at both 15 days (22.3%, *P* = .031) and 17 days (36.5%, *P* = .0086) after the start of treatment (Fig. 5E). A significant increase of 8.5 days in median survival was found when administering 4 Gy TBI before nilotinib compared with 4 Gy TBI after nilotinib (*P* = .0036, Fig. 5F). Results show a substantial survival benefit when performing 4 Gy TBI before administering nilotinib.

## Discussion

In this study, compartmental modeling and multiphoton microscopy were combined to create QMPM, a single-platform method to both measure BMV functional characteristics and assess the pharmacokinetics of fluorescent therapeutic analogs. Assessments of mice bearing ALL revealed increased permeability, altered K_trans_, increased V_ec_, reduced vascular perfusion, increased mean vessel density, and reduced mean vessel diameter. ALL-induced alterations reduce cellular drug uptake of therapeutics. As hypothesized, LDRT increased BMV perfusion and enhanced therapeutic delivery to ALL cells in the bone marrow. Consequently, 4 Gy TBI before the administration of nilotinib significantly increased the survival of mice bearing ALL compared with treatment with TBI after nilotinib administration. These results demonstrate the ability of QMPM to characterize and optimize vasculature function for combination treatments of hematopoietic malignancies.

QMPM has the ability to model the transport of therapeutic analogs in the local tissue and vascular compartments to obtain K_trans_ and V_ec_. Changes in the vascular transport of molecular agents occur early in the progression of malignant disease, making V_ec_ and K_trans_ useful as potential biomarkers and for assessing drug or oxygen delivery.^37,38^ Limited work has been done with microscopy to perform tissue compartmental modeling for the identification of vascular permeability.^16,23^ Moreover, to our knowledge there have been no successful attempts using time-lapsed microscopy data to perform compartment modeling and obtain both K_trans_ and V_ec_ for the local vasculature. Differences in tracer concentration between the local vasculature and arterial blood may be present in poorly perfused tissues.^14,28^ In such a case, QMPM images of the local vasculature will represent the local tracer transport rather than the typical transport from arterial blood to whole tissue. However, in the case of well-perfused tissue, as is likely in this work, arterial and local vascular blood concentration measurements will be relatively similar. QMPM can be used in many different tissue types with many fluorescent agents, making it applicable to a wide variety of biological applications, such as the delivery of nutrients, oxygen, and therapeutics to the bone marrow.

At low to moderate leukemic burden, dextran-based K_trans_ is increased compared with that in healthy mice, likely because of increased BMV permeability, enabling faster dextran leakage through the endothelial wall. Increased K_trans_ values in low to moderate ALL burden may indicate a therapeutic window of enhanced delivery for ALL antibody therapies, such as those targeting CD19 or CD22, which are similar in size to 150KDa dextran.^39,40^ However, differences in vascular transport mechanisms of dextran and therapeutic antibodies make further assessments of antibody delivery kinetics necessary.^41^ In contrast to 150kDa dextran, small molecular agents such as Hoechst dye and daunorubicin are able to easily cross the vessel wall of both healthy and ALL-burdened BMV. This results in decreased cellular uptake of small-sized therapeutics with the onset of ALL, due in part to reduced blood perfusion.

Previous reports have shown arterial and sinusoidal blood vessels to be linked with phenotypes observed in healthy mice. Small-diameter, fast-flow BMV correlates with arterial vessels, whereas large-diameter, slow-flow BMV correlates with sinusoidal vessels and venules for healthy mice.^18,23^ With ALL vascular remodeling, the structure of the healthy BMV is lost because of unchecked angiogenesis, creating semicollapsed, immature, permeable vessels. This results in poor delivery of therapeutic agents, oxygen, and nutrients through the BMV. Increased hypoxia in the bone marrow has been reported with multiple hematological malignancies and is likely due to similar vascular alterations as reported in this work.^42^ Additionally, early preservation of the healthy BMV by the inhibition of vascular remodeling in vivo has been shown to give a survival advantage for leukemic mice treated with chemotherapy, suggesting drug delivery is hampered by the onset of malignancy.^5^ A restoration of BMV perfusion and reduction of high disease burden are crucial to improve the delivery of therapeutics to the bone marrow.

This study suggests a possible new role for LDRT in restoring the biophysical function of the malignant BMV for combination therapies. Neoadjuvant LDRT treatments to ALL-burdened mice enhance BMV perfusion, increase delivery of therapeutics, and improve treatment efficacy. Similar RT-induced vascular alterations in solid tumors have been reported and may stem from nitrous oxide–dependent vasodilation.^43^ Our results demonstrate the ability of LDRT to actively alter the BMV and indicate a possible new treatment approach for hematologic malignancies. Although recently developed total marrow irradiation is capable of delivering highly conformal treatment to the bone marrow^9,44,45^ or to smaller regions of high disease burden,^46^ it is often used in conjunction with high-dose regimens to enhance cell killing.^47^ In contrast, LDRT can provide both cell killing and vascular modulation to enhance therapeutic delivery in desired regions.

With the wide variety of therapeutics that can be used with LDRT, consideration should be given to select synergistic treatment approaches. LDRT treatments may be particularly effective for vasculature targeting therapeutics, in which delivery depends only on vascular blood perfusion to endothelial cells. Vascular disrupting agents frequently elicit off-target toxicity,^48^ and therapies designed for vascular normalization frequently lead to pathway resistance, making combination therapy with RT desirable.^49,50^ Vascular targeted therapeutics show synergistic effects after low- to moderate-dose RT in solid tumor models, allowing for reduction in drug delivery without compromising treatment efficacy.^51^ Immunotherapy has also shown synergy with LDRT in solid tumor models.^52^ Additionally, LDRT for hematopoietic malignancies can be explored with more traditional therapeutic agents that elicit off-target toxicity or have synergistic effects.^53,54^

Although QMPM is useful to understand therapeutic delivery and microscopic vascular changes in the bone marrow, it is limited to preclinical studies only. Noninvasive macroscopic imaging such as dynamic contrast-enhanced MRI will need to be used to investigate the effects of LDRT on therapeutic delivery and the BMV for clinical translation. However, the resolution of macroscopic imaging makes it unsuitable for observing single vessel characteristics. Histology will need to be performed to observe the morphology of the BMV. Additionally, neither histology nor macroscopic imaging modalities will be able to measure the functional single-vessel parameters that can be observed with QMPM. In the future, correlation of QMPM’s microscopic imaging with macroscopic imaging will help in better understanding and interpreting the underlying BMV changes during macroscopic imaging for clinical translation.

## Conclusions

LDRT-induced alterations to functional BMV characteristics are one example of the many possible applications for QMPM. Simultaneous measurements of both functional single-vessel BMV characteristics and therapeutic pharmacokinetics using QMPM are able to reveal and optimize oxygen, nutrient, and drug transport for many disease models and tissues. In the context of ALL, such measurements are crucial to understanding LDRT treatment-induced BMV alterations and their effects on treatment outcome. Future work exploring LDRT will investigate the effects of timing, dose, and dose fractionation on the treatment of hematologic malignancies. QMPM measurements will be essential in observing the BMV for LDRT-based combination therapies.

## Supplementary Material

mmc7

mmc6

mmc6fig

mmc5

mmc5fig

mmc4

mmc4fig

mmc3

mmc3fig

mmc2

mmc2fig

mmc1

mmc1fig

## Figures and Tables

**Table 1:** ALL cellular uptake of Hoechst and daunornbicin

	2 Gy tLDRT (n = 3 mice)		4 Gy tLDRT (n = 3 mice)	
	Abscopal	Targeted Tissue	Abscopal	Targeted Tissue
Mice 9 d post-ALL injection				
Femur ALL engraftment (%)	28.5 ± 6.3	12.2 ± 1.7	34.5 ± 0.8	16.4 ± 2.7*
Calvarium ALL engraftment (%)	35.4 ± 7.0	28.1 ± 3.6	25.2 ± 2.1	18.4 ± 5.3
Hoechst+ cells in ALL fraction in femur (%)	95.3 ± 0.9	96.4 ± 0.7	92.0 ± 1.1	93.9 ± 1.0
Hoechst mean fluorescent intensity of	12.0 ± 1.0	13.4 ± 0.9	10.4 ± 0.5	12.8 ± 0.6*
ALL cells in femur (AU)				
Hoechst+ cells in ALL fraction in calvarium (%)	63.5 ± 3.0	88.3 ± 3.6^†^	72.6 ± 7.2	84.5 ± 0.7
Hoechst mean fluorescent intensity of ALL cells in calvarium (AU)	10.4 ± 0.7	12.3 ± 0.9	10.4 ± 0.3	13.7 ± 1.2
Mice 7 d post-ALL injection				
Femur ALL engraftment (%)	26.8 ± 3.8	6.4 ± 1.3*	35.3 ± 11.4	5.4 ± 2.6
Daunorubicin+ cells in ALL fraction in femur (%)	72.3 ± 1.8	90.8 ± 2.3^†^	78.0 ± 3.4	93.5 ± 3.7*
Daunorubicin mean fluorescent intensity of ALL cells in femur (AU)	27.0 ± 1.1	37.8 ± 2.5*	27.9 ± 2.8	48.0 ± 10.4

*Abbreviations:* ALL = acute lymphoblastic leukemia; tLDRT = targeted low-dose radiation therapy.

* Indicates Welch 2-sided *t* test value of *P* < .05 when comparing matching abscopal and targeted tissue regions.

† Indicates Welch 2-sided *t* test value of *P* < .01 when comparing matching abscopal and targeted tissue regions.
