# Supplementary material for: Biophysical Characterization of the Leukemic Bone Marrow Vasculature Reveals Benefits of Neoadjuvant Low-Dose Radiation Therapy
Source: Int J Radiat Oncol Biol Phys. Author manuscript; Available in PMC 2021 Jan 1. (PMC7736317; doi:10.1016/j.ijrobp.2020.08.037)
Supplement: mmc7 [file NIHMS1623682-supplement-mmc7.docx]

## Appendix A: Mice, drug treatments, and survival study

Eight-to-ten week-old female mice were used in experiments pertaining to endothelial cell analysis, QMPM tile image acquisition, and QMPM vessel morphology analysis. Experiments pertaining to vascular compartmental modeling, dextran well counter fluorescence, total bone marrow cell counts, and Hoechst dye uptake were performed using 7-10 week-old female mice. Intravascular cellular velocity measurements were performed using 7-12 week-old female mice. Daunorubicin QMPM imaging and daunorubicin cellular uptake assays were performed using 10-12 week-old male mice at moderate ALL burden (10-50%). Survival study experiments were performed using 8 week-old female mice.

Mice for survival studies were monitored for disease progression by peripheral blood sampling. Survival study treatment intervention started seven days after ALL injection when ALL was detectable in the blood of all the mice. Nilotinib treatment was administered using oral gavage at 50 mg/kg suspended in 100 µl of 79% PBS and 21% DMSO. Mice not receiving nilotinib were administered 100 µl of 79% PBS and 21% DMSO on matching days.

## Appendix B: Mouse surgery, multiphoton image acquisition, and image display

For calvarium imaging, mice were anesthetized initially with 3% isoflurane at a flow rate of 2 L/min, and then maintained with 1.5% isoflurane at a flow rate of 1.5 liters per minute. A stereotactic apparatus with a bite bar was utilized to keep the mouse stable during surgery. The skin and membrane above the calvarium was removed, and a custom titanium head plate with an inner diameter of 8 mm was fixed to the calvarium using Alpha-Dent Polycarboxylate Cement (Dental Technologies, Lincolnwood, IL). The head plate was then inserted into a custom built heated stage maintained at 37°C allowing for easy viewing of the mouse calvarium. For imaging, a Prairie Ultima multiphoton microscope (Bruker Corporation Billica, MA) was used. Fluorescent excitation was performed using a Chameleon Ultra II tunable Ti:Sapphire laser with 140 femtosecond pulses (Coherent, Santa Clara, CA). An Olympus XLUMPlanFL 20x objective (1.00 NA water objective) was used for image acquisition of all images. QMPM imaging was primarily performed on the frontal bone region of the calvarium, however 2-3 mice with high leukemic burden were imaged on the superior parietal bone near the coronal suture, as ALL-induced bone degradation made imaging difficult on the frontal bone region. Simultaneous four channel acquisition was performed at 660/40 (far red), 595/50 (red), 525/50 (green), and 460/50 (blue) for visualization of ALL, blood pool agents and bone-collagen. Data acquisition using the Prairie Ultima microscope was handled by Prairieview 5.3 software and imaging was performed at room temperature.

Excitation for QMPM was performed at 900 nm for GFP+ ALL fluorescence, TritC dextran fluorescence, and Qtracker™ 655 Vascular Label fluorescence (acquired primarily in the green, red, and far red channels respectively). Additional excitation was performed at 840 nm and 960 nm for visualization of both daunorubicin fluorescence (red channel) and GFP^+^ ALL fluorescence (green channel), respectively. The second harmonic generation image of the collagen in the bone was performed at 900 nm and 960nm and was detected in the blue channel. Color channel windows were adjusted individually in Fiji/ImageJ to improve image display quality. Individual color window adjustments were identical between sets of comparative images.

Image subtraction and cellular segmentation was performed using Cell Profiler. Image subtraction was used for quantitative GFP+ ALL and static daunorubicin fluorescence analysis as well as for the presentation of all images except time-lapsed daunorubicin and time-lapsed dextran image sets. Image subtraction minimized signal due to background fluorescence, which came from auto-fluorescent cells and collagen and is present in several acquisition channels. For image display and quantification of GFP+ ALL fluorescence in images acquired at 900nm excitation, auto fluorescence from blue and red channels were multiplied by a scalar and subtracted from the green channel to eliminate signal from both collagen and auto-fluorescent cells respectively. For image display and quantification of daunorubicin and GFP+ ALL fluorescence in single time-point image sets, image subtraction was performed by multiplying the 840 nm excitation blue channel signal by a scalar and subtracting from the 960 nm excitation green channel and 840 nm excitation red channel to identify GFP^+^ ALL and daunorubicin fluorescence, respectively. Healthy mice without daunorubicin injections were used to determine image subtraction scalars.

***Appendix C:*** ***Image tiling, vascular morphology measurements, and intravascular cellular velocity measurements***

Tiled images were acquired by collecting approximately a 5x4 grid series of overlapping z-stack images. The images were overlapping by 15% with a resolution of 512 by 512 pixels and a z-slice spacing of 15 µm. Tiled images were stitched together using a Fiji/ImageJ grid collection/stitching plug-in. Vascular diameter and the number of vessel branches per area were quantified using Fiji/ImageJ. Vessel cellular velocities were measured after injection of either 150 kDa TRITC dextran or Qtracker™ 655 Vascular Label. Cells in the bloodstream could be visualized as shadows passing through the blood plasma. Repeated line scans were taken transaxially on the center of blood vessels with frequencies of approximately 0.4 to 3 kHz. Line scans obtained time-space images with cells moving through the vessels visualized as dark diagonal streaks. The average slope of 3 separate cells was found from time-space images using Fiji/ImageJ to calculate intravascular cellular velocity for a given vessel.

## Appendix D: Time-lapsed imaging and compartmental modeling

Time-lapsed single or z-stack images of dextran leakage into the tissue were taken initially with 6-15 second intervals, increased to approximately 60 seconds after peak tissue concentration had been reached. Image drift over time was accounted for by co-registration of time frames in Matlab®. To identify blood and tissue compartment regions, time-lapsed images of dextran were cropped to in focus regions. The first frame after dextran had completely perfused the vasculature was selected to perform vascular segmentation. Images were smoothed with a median filter of four pixels in diameter and an Otsu’s thresholding algorithm was applied to segment the vasculature from the tissue and background. If a single Otsu’s thresholding algorithm was not adequate to properly identify the vasculature, the first thresholded region was removed from the smoothed image and a second Otsu’s thresholding algorithm was applied to identify the remaining vascular regions. After vascular segmentation, an image frame after the dextran tissue concentration had reach maximum (typically the 100^th^ frame) was selected. A Gaussian filter of four pixels in diameter was applied to the image and the vascular regions found earlier were removed from the image. Another Otsu’s threshold was applied to segment the regions of tissue uptake from the background. Since spatial overlap in the dextran fluorescent signal of blood and tissue compartments occurred primarily near boundary pixels in the regions of interest, four and eight pixel erosions to the edges of the vascular and tissue regions of interest were performed respectively. This better isolated dextran fluorescence from tissue and blood compartments. After applying Otsu’s thresholding, regions of obvious segmentation mismatch were identified and removed, as such regions would significantly affect quantification accuracy. In general, needed removals were less than 10% of the total segmented volume. For daunorubicin compartment identification, blood and tissue compartments were identified in similar fashion as dextran compartments, while the daunorubicin cellular compartment was identified using simple manual selection of representative cellular signal at a late time point after blood and tissue clearance.

Dextran fluorescent intensity in blood and tissue compartments was quantified after spectral unmixing of GFP+ ALL signal bleed through into dextran signal calculated using Eq. (D1).

$I_{D}\left( t \right)=\frac{R\left( t \right)-R_{B}*\frac{G(t)}{G_{B}}}{A}$ [1]

Where R(t) and G(t) are the total red and green channel signal intensity at time t for the region of interest. R_B_ and G_B_ are the background signal intensities prior to dextran injection for the red and green channels, respectively. A is the area of the region of interest being analyzed. The equation for spectral unmixing was used to account for photobleaching of GFP+ ALL cells during time-lapsed imaging. Daunorubicin time-lapsed quantification was performed using pre-injection signal background subtraction rather than spectral unmixing, as minimal amounts of ALL signal was present in the daunorubicin channel.

Dextran leakage from tissue and blood regions of interest was modeled using Eq. (D2).

$\frac{dC_{t}}{dt}=\left( K_{trans} \right)(C_{p}(t)-v_{ec}{*C}_{t}(t))$ [2]

Where $C_{p}(t)$ is the capillary plasma tracer concentration and $C_{t}$ is the tracer tissue concentration. A value of 0.4 for the hematocrit was used to convert blood concentration to $C_{p}(t)$. $C_{p}(t)$ was modeled by a summation of two separately weighted exponential decay curves and fit to each individual animal’s dextran fluorescent intensity time-curves for the blood compartment. Curve fitting was performed in Matlab using the lsqnonlin() function. Eq. (D2) was solved for by substituting the blood plasma equation and setting the initial tissue concentration equal to zero to obtain Eq. (D3).

$C_{t}\left( t \right)=K_{trans}\sum_{i=1}^{n} \left[ \left( \frac{A_{i}}{\left( {K_{trans}}/{\nu_{ec}} \right)-\mu_{i}} \right)\left( e^{-\mu_{i*}t}-e^{-\left( {K_{trans}}/{\nu_{ec}} \right)*t} \right) \right]$ [3]

Where $A_{i}$ and $\mu_{i}$ are the scaling coefficient and attenuation coefficient for the i^th^ exponential plasma concentration curve, respectively. $A_{i}$ and $\mu_{i}$ are used to fit equation 3 to dextran fluorescent intensity time-curves for the tissue compartment, solving for K_trans_ and ν_ec_. The fitting was performed from a range of starting conditions across the acceptable boundary conditions to ensure the error function was globally minimized.

It can be shown that K_trans_ is approximately equal to the average vascular permeability times the average vascular surface area per volume of tissue when the majority of first pass dextran through the BMV remains in the vasculature and does not entirely permeate into the tissue bed. Vascular permeability was calculated by dividing K_trans_ by vessel surface area per imaging volume. The length and diameter of the lumen for each vessel was measured manually in Fiji/ImageJ, and the respective surface area was calculated by approximating vessels as cylinders. The amount of available calvarium marrow space in each imaging area was accounted for by observation of the second harmonic generation image of the collagen in the bone.

## Appendix E: Blood pressure measurements

Mouse blood pressure was recorded in mice using the BP-2000 Blood Pressure Analysis System (Visitech Systems). Thirty successive tail cuff measurements were taken on each day. The first 10 measurements were discarded to allow time for mice to grow accustomed to the tail cuff. Successful measurements from the last 20 measurements were averaged together to obtain results.

## Appendix F: CT image acquisition and LDRT treatments

CT skull imaging was performed with 40 kVp, 1 mA, and a 100 µm pixel resolution. Treatment was performed at 255 kVp and 13 mA. For calvarium treatment, the edge of an anterior 10 mm square beam treated the calvarium with a soft-tissue-equivalent dose of 2 Gy or 4 Gy. This resulted in a dose of approximately 5 Gy or 10 Gy to treated calvarium bone respectively as calculated using the Monte Carlo simulated treatment planning software on the small animal image guided irradiation system. This dose enhancement is largely due to the increased effective atomic number of bone compared to soft tissue. At an effective treatment photon energy of 78.8keV there will be increased dose absorption in bone compared to soft tissue due to photo-electric based dose absorption. The beam was placed 200 µm to the mouse’s right side from the sagittal suture and completely covered the parietal and frontal bones on the mouse’s left side. The left femur was treated with 10 mm square anterior to posterior and posterior to anterior beams. Dose was normalized to 2 Gy or 4 Gy 2 mm below the surface of the skull for parietal beam and 2 Gy or 4 Gy within the marrow for the femur beams.

## Appendix G: Flow cytometry

For endothelial cell analysis, the tibias and femurs from mice and were crushed and incubated with collagenase (3 mg/ml, Sigma Aldrich, St Louis, MO) for 45 min at 37°C to liberate the leukemic and bone marrow microenvironment cells from the bone surface. Cells were incubated with ACK lysis buffer (Gibco) for 5-10 minutes, and washed with PBS. The cells were filtered through a 40 μm filter and centrifuged at 300xg for 5 min with PBS (0.1%BSA) solution at 4°C. The cells were then blocked with CD16/32 blocking antibody, followed by staining with CD45 (PE/Cy5, clone 30-F11), Ter119 (PE/Cy5, Ter119), CD31 (PE/Cy7, clone 390) (Bio Legend, San Diego), and endomucin (A660, eBioV.7C7 ebioscience) antibodies and with AmCyan viability dye for 30-45 min at 4°C in dark. After staining, cells were washed in PBS (0.1% BSA). Stained cells were analyzed on FACSARIAIII (BD bioscience, Franklin Lanes, NJ) and analyzed in live (AmCyan-), non-leukemic (GFP-) CD45-Ter119-CD31+ fraction. Flow cytometry analysis was performed using FlowJo 10.5.3 software (FlowJo, Ashland, OR).

For Hoechst dye uptake, tLDRT-treated and abscopal femur and calvarium samples were crushed in PBS (0.1%BSA) solution. Cells suspensions were filtered through a 40 μm filter and centrifuged at 300xg for 5 min at 4°C, then incubated with ACK lysis buffer for 5-10 minutes to remove red blood cells. The cells were analyzed for Hoechst uptake in both the total cell and GFP+ ALL cell populations using the BD Fortessa (BD bioscience, Franklin Lanes, NJ) on the same day.

For daunorubicin uptake, tLDRT-treated and abscopal femur samples were crushed in PBS (0.1%BSA) solution. Cells suspensions were filtered through a 40 μm filter and centrifuged at 300xg for 5 min at 4°C, then incubated with ACK lysis buffer for 5-10 minutes to remove red blood cells. The cells were stained with AmCyan viability dye (1 µg/ml) for 15 min and washed with PBS. Cells were analyzed on AmCyan- (live) GFP+ (leukemic) cell population using BD FACSARIAIII sorter 2-3 hours post-harvest and were kept on ice throughout the staining process.

To monitor GFP+ ALL progression, 5-10 µL of peripheral blood was taken from the tail vein of mice. Samples were incubated with to ACK lysis buffer for 5-10 minutes to remove red blood cells, then washed with PBS and read using the BD Fortessa within 24 hours. Measurements of GFP+ ALL femur engraftment were analyzed in similar fashion after femur samples were crushed in PBS (0.1%BSA) solution, filtered through a 40 μm filter, and centrifuged at 300xg for 5 min at 4°C.

## Appendix H: Dextran well plate readings

Dextran femur fluorescence was measured in imaged mice that were euthanized 50 minutes after 150kDa TritC dextran injection. Femurs were crushed in 7 ml of PBS (0.1%BSA) solution and centrifuged at 300xg for 5 minutes at 4°C. A total of 200 µl of supernatant was removed and read using a FilterMax F5 Multi-Mode Microplate Reader (Molecular Devices San Jose, CA). Well plate readings were linear over the read range (data not shown).


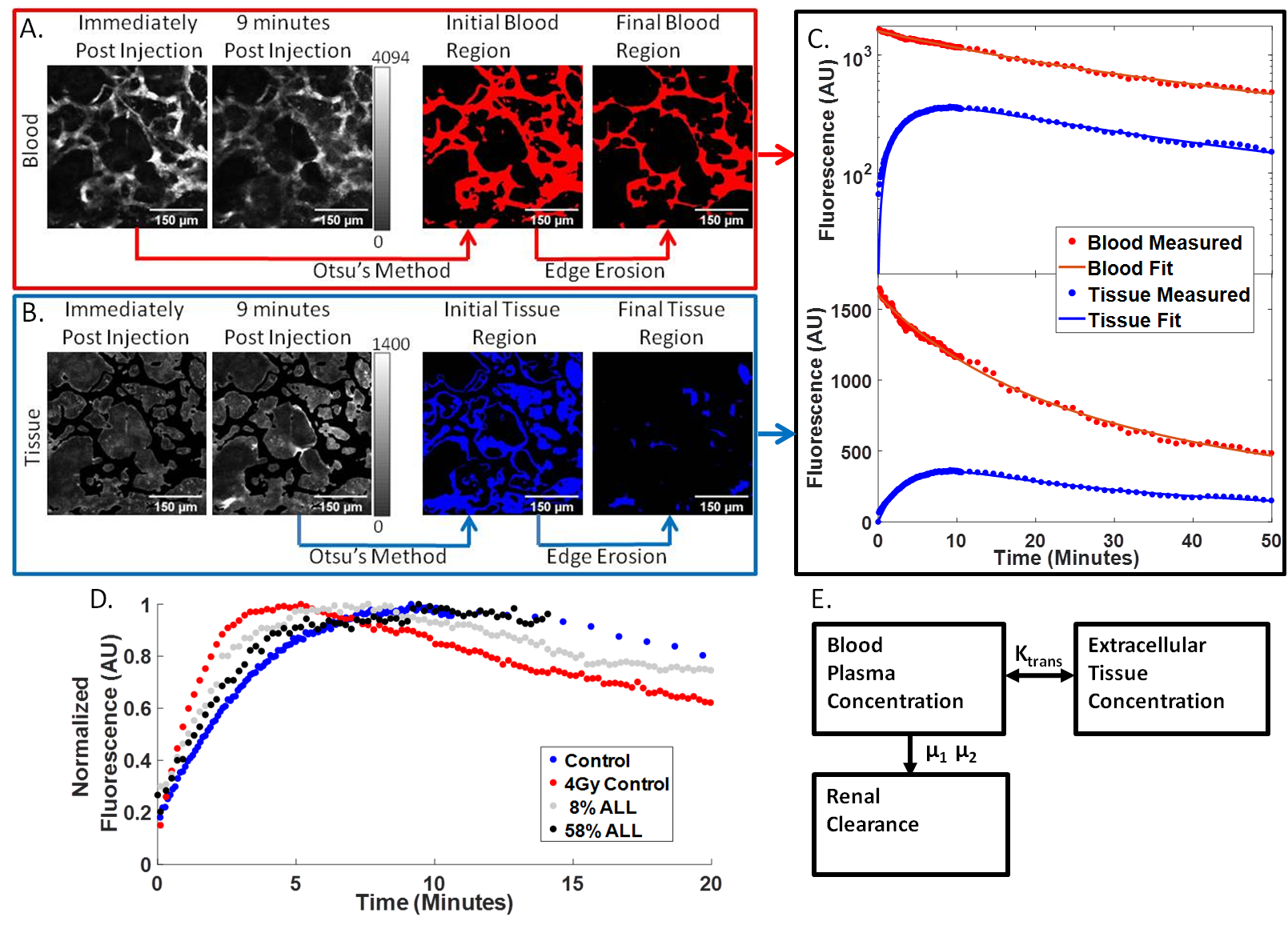


**Figure E1.**

**Segmentation of time-lapsed dextran imaging and compartmental modeling**

The segmentation schemas for (A) the blood compartment and (B) the tissue compartment for 150kDa TritC dextran compartmental analysis are shown. Briefly, the time-lapsed images are co-registered, and Otsu’s method is used on images at an early time point to segment the vascular regions. To segment the tissue region, a later time point image is selected after dextran leakage into the tissue. The previously segmented vascular region is removed, and Otsu’s method is used to segment the tissue regions. Edge erosion is applied to the segmented regions to better isolate dextran fluorescent signal in the blood and tissue compartments. Scale bars indicate the imaging window range for displayed early and late blood and tissue image sets. (C) The corresponding blood and tissue compartment time-lapsed dextran fluorescent intensity from the segmented regions is displayed in linear and log scale with corresponding fitting function curves. (D) A plot of the normalized time-lapsed dextran fluorescent intensities from the segmented tissue compartments of a healthy control mouse, a non-leukemic 4 Gy tLDRT-treated mouse, a mouse with 8% ALL femoral bone marrow engraftment, and a mouse with 58% ALL femoral bone marrow engraftment. Matching time-lapsed videos and matching absolute time-lapsed data is displayed in Video E2-4 and Figure 2H. (E) A diagram of the compartment model used to obtain compartmental modeling parameters from time-lapsed dextran images is shown.


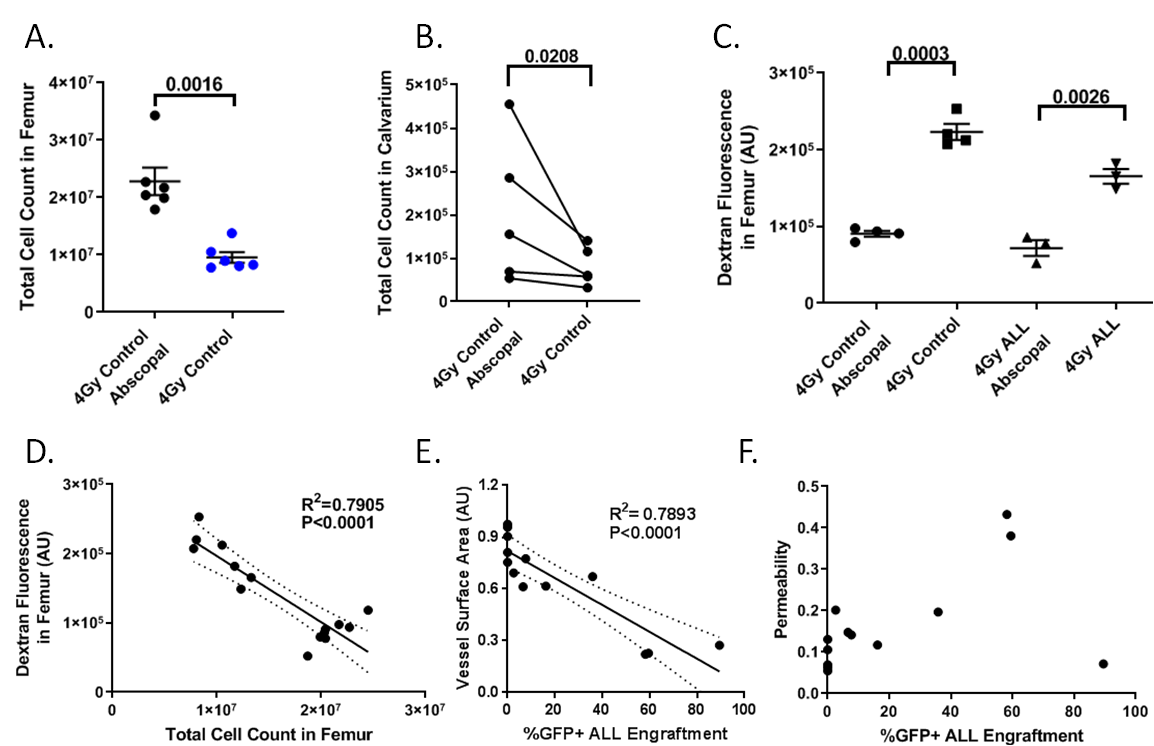


**Figure E2.**

**Compartmental modeling validation and parameters**

(A) Total number of bone marrow cells in crushed 4 Gy tLDRT-treated and abscopal femur samples from non-leukemic mice (n=7, 6 mice per group respectively). (B) Total number of calvarium bone marrow cells in crushed tLDRT-treated and abscopal calvarium regions of non-leukemic mice with a two sided paired t-test calculation (n=5 mice). (C) Dextran fluorescence of crushed femur supernatants from non-leukemic abscopal, non-leukemic 4Gy tLDRT-treated, ALL abscopal, and ALL 4Gy tLDRT-treated single femurs (n=4, 3 non-leukemic mice and mice bearing ALL respectively). (D) The number of total bone marrow cells in crushed single femur samples versus dextran fluorescence of crushed femur supernatants is shown (n=15 single femurs). Abscopal and treated femurs taken from LDRT-treated non-leukemic mice and LDRT-treated mice bearing ALL are shown. The corresponding R-squared value and best fit line are also displayed. (E) Vessel surface area versus GFP+ ALL femur bone marrow engraftment with the corresponding R-squared value and best fit line are plotted for untreated mice analyzed with QMPM compartmental modeling (n=13 mice). (F) ALL femoral bone marrow engraftment plotted versus vascular permeability for untreated mice analyzed with QMPM compartmental modeling (n=13 mice).


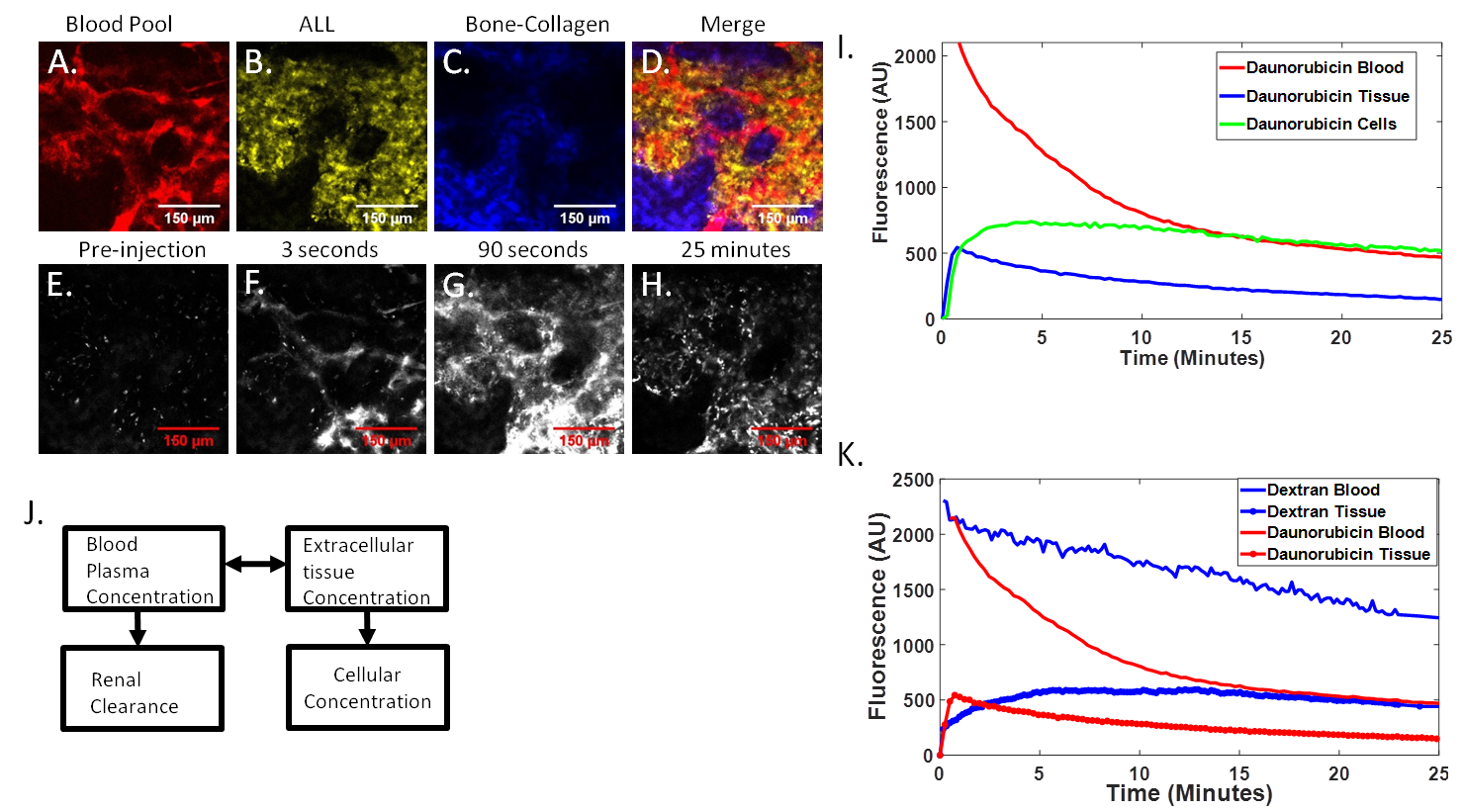


**Figure E3.**

**Compartmental modeling of daunorubicin**

QMPM images of ALL burdened mouse calvarium showing (A) Qtracker™ 655 vascular blood pool fluorescence, (B) GFP+ ALL fluorescence, (C) the second harmonic generation image of the collagen in the bone, and (D) a merged image are shown for a daunorubicin-injected mouse. Time-lapsed imaging of daunorubicin fluorescence (E) before, (F) three seconds post, (G) 90 seconds post, and (H) 25 minutes post daunorubicin injection are shown. A clear distinction between blood, tissue, and cellular compartments can be seen. (I) Plots of fluorescent intensity versus time for contoured blood, tissue, and cellular compartments are shown. (J) A representative image of a three tissue compartment model applicable to daunorubicin is shown. (K) A comparison of fluorescent intensities of blood and tissue compartments for daunorubicin and 150kDa TritC dextran in separate mice bearing ALL is shown.

**
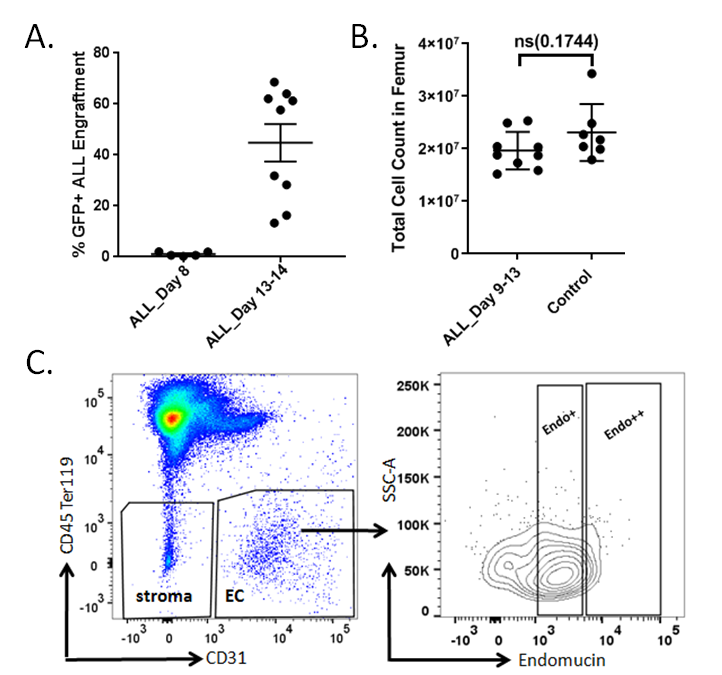
**

**Figure E4:**

**Endothelial cell flow cytometry analysis**

(A) Corresponding GFP+ ALL engraftment for mice analyzed for endothelial cell flow cytometry analysis in Figure 3F-G (n=5, 9 mice per group respectively). (B) Total number of cells from the bone marrow of crushed single femurs for untreated healthy control mice and untreated mice 9-13 days post injection of ALL (n=9,7 mice per group respectively). (C) The flow cytometry gating scheme for CD45Ter119- CD31+ endothelial cell population and high endomucin subpopulation is shown.

**Figure E5:**

**BMV measurements of vascular structure and blood perfusion in mice bearing ALL**

Merged color images of the calvarium of (A) mice bearing ALL and (C) control mice. Green is GFP+ ALL fluorescence, red is Qtracker™ 655 blood pool fluorescence, and blue is the second harmonic generation of collagen in the bone. Vascular blood pool images with corresponding blood velocity mapping of upstream to downstream vessels labeled 1-11 are displayed for (B) ALL burdened vessels and (D) healthy vessels. Mice bearing ALL were imaged 8 days post ALL injection. (E) Intravascular cellular velocity and (F) vessel diameter measurements from labeled vessels in B and D show the loss of upstream, fast-flow, small-diameter vessels and downstream, large-diameter, slow-flow vessels in mice bearing ALL. Plots of cell velocity versus diameter for (G) ALL burdened vessels eight days post ALL injection and (H) healthy control vessels (n=3 mice per group, 10 vessel measurements per mouse).


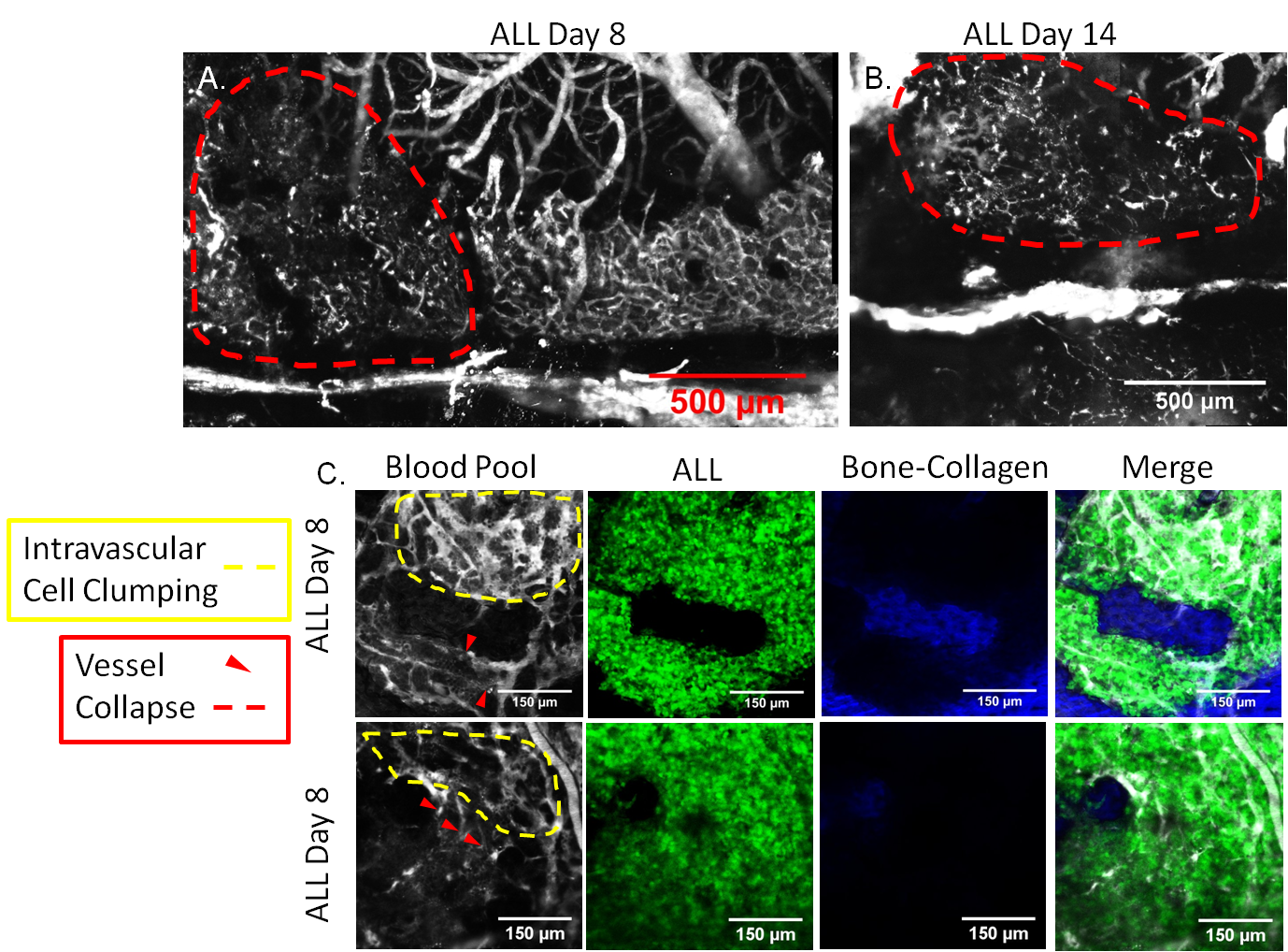


**Figure E6:**

**Vascular collapse and intravascular cellular accumulation in mice bearing ALL**

Tiled QMPM images of Qtracker™ 655 vascular blood pool fluorescence in the calvarium of ALL-transplanted mice at (A) 8 days and (B) 14 days post ALL injection. Red dashed lines indicate regions of vascular collapse. (C) Merged composite images of Qtracker™ 655 blood pool fluorescence (gray), GFP+ ALL fluorescence (green), and the second harmonic generation image of the collagen in the bone (blue) for ALL injected mice 8 days post injection. Red triangles and red dashed lines indicate specific vessel collapse and regions of vascular collapse, respectively. Yellow dashed lines indicate regions of accumulated non-moving cells in the vascular BMV.


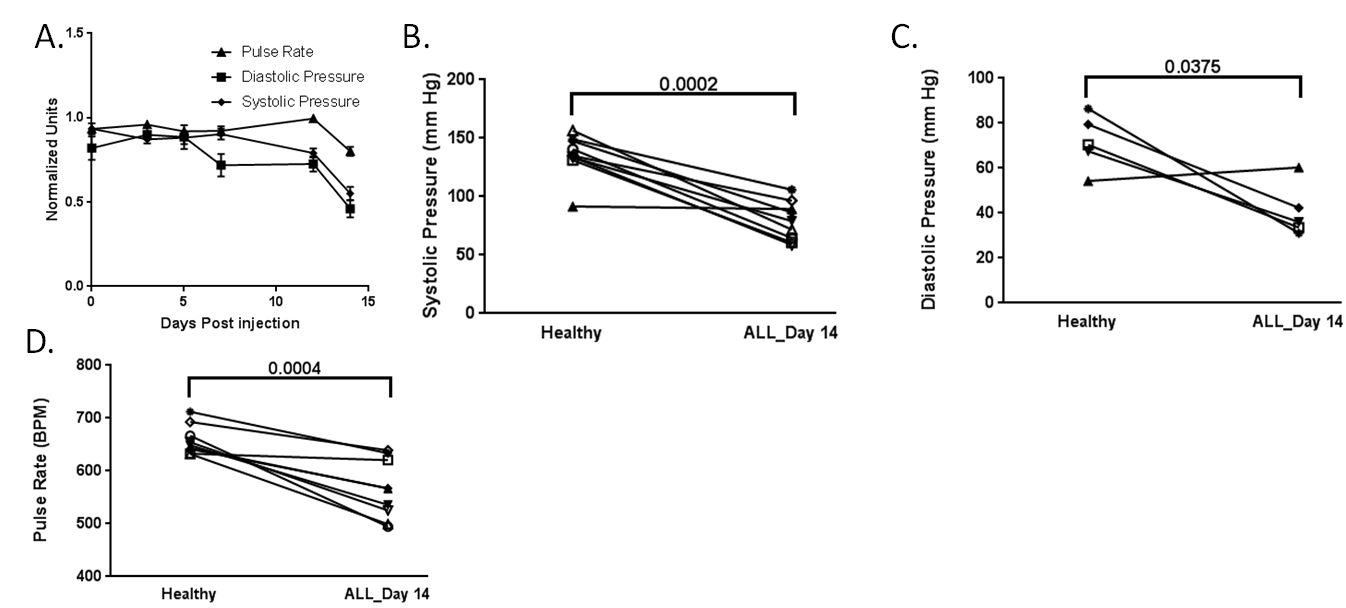


**Figure E7:**

**Changes in systemic blood pressure with the onset of ALL**

(A) A normalized plot of mouse pulse rate, diastolic blood pressure, and systolic blood pressure before ALL injection and 3, 5, 7, 12, and 14 days following ALL injection. (B-D) Plots of systolic blood pressure, diastolic blood pressure, and pulse rate in mice before and 14 days after receiving ALL injection with two-sided paired t-test significance values.

**
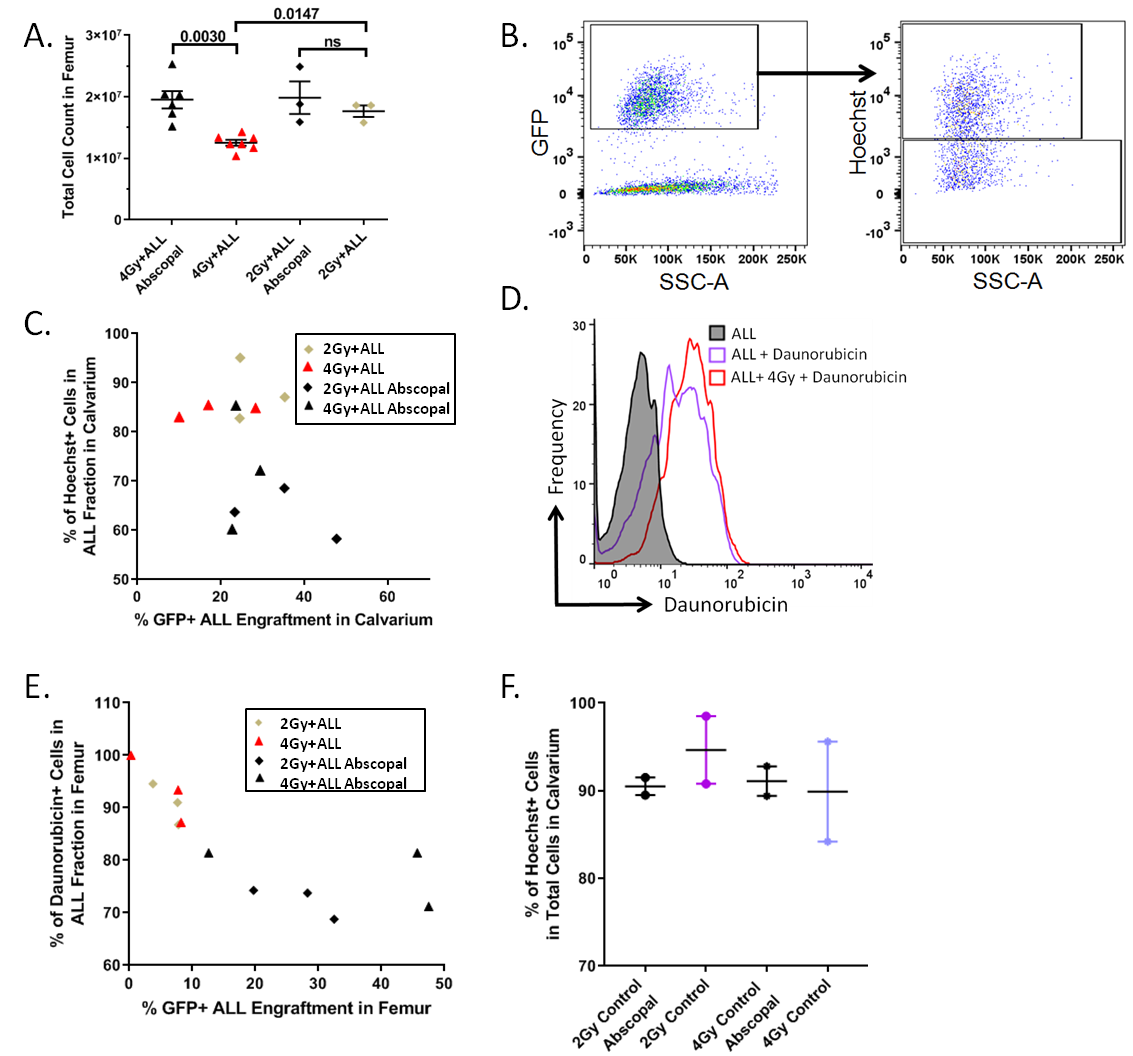
**

**Figure E8:**

**Hoechst and daunorubicin uptake gating and analysis**

(A) Total cell counts for 4Gy LDRT-treated and 2 Gy LDRT-treated crushed femur bone marrow samples from mice bearing ALL (n=6,3 mice per group respectively, one abscopal and one treated femur region per mouse). Treated regions are plotted next to their corresponding abscopal regions for comparison (B) A representative gating scheme for Hoechst+ GFP+ and Hoechst- GFP+ ALL populations. (C) A plot of the percentage of GFP+ ALL cells in the calvarium stained positive for Hoechst versus total calvarium ALL engraftment in tLDRT-treated and abscopal calvarium regions (n=3 mice per group, one abscopal and one treated calvarium region per mouse). (D) Representative histograms of daunorubicin fluorescence in ALL cells from crushed femur bone marrow samples. (E) A plot of the percentage of GFP+ ALL cells labeled positive for daunorubicin is shown for 4 Gy tLDRT–treated regions, 2 Gy tLDRT–treated regions, and corresponding abscopal regions (n=3 mice per group, one abscopal and one treated femur per mouse). (F) A plot of the percentage of total cells labeled positive for Hoechst in 2 Gy tLDRT-treated, 4Gy tLDRT-treated and corresponding abscopal calvarium regions for non-leukemic mice (n=2 mice per group, one abscopal and one treated calvarium region per mouse).

**Video E1:**

**Three-dimensional tile image of mouse bearing ALL**

A multiphoton microscopy-based three-dimensional tiled image play-through of the calvarium of a mouse 4 days post injection of GFP+ ALL. The z spacing between images is 15 µm. Blue is the second harmonic generation image of the collagen in the bone, green is GFP+ ALL fluorescence, and red is Qtracker™ 655 blood pool fluorescence.

**Video E2:**

**Time-lapsed dextran imaging of healthy control mouse**

Time-lapsed imaging of 150kDa TRITC dextran fluorescence (gray) during dextran injection into a healthy control mouse is shown. Time post injection is displayed. The second harmonic generation image of the collagen in the bone (blue) is displayed to show bone marrow compartments within the calvarium. Matching time-lapsed tissue compartment fluorescent intensity from this mouse is shown in Figures 2H and S1D.

**Video E3:**

**Time-lapsed imaging of non-leukemic 4Gy tLDRT-treated mouse**

Time-lapsed imaging of 150kDa TRITC dextran fluorescence (gray) during dextran injection into a non-leukemic 4Gy tLDRT-treated mouse is shown. Time post injection is displayed. The second harmonic generation image of the collagen in the bone (blue) is displayed to show bone marrow compartments within the calvarium. Matching time-lapsed tissue compartment fluorescent intensity from this mouse is shown in Figures 2H and S1D.

**Video E4:**

**Time-lapsed imaging of mouse with 8% ALL femoral bone marrow engraftment**

Time-lapsed imaging of 150kDa TRITC dextran fluorescence (gray) during dextran injection into a mouse with 8% ALL femoral bone marrow engraftment is shown. Time post injection is displayed. The second harmonic generation image of the collagen in the bone (blue) is displayed to show bone marrow compartments within the calvarium. Matching time-lapsed tissue compartment fluorescent intensity from this mouse is shown in Figures 2H and S1D.

**Video E5:**

**Time-lapsed imaging of daunorubicin**

Time-lapsed imaging of daunorubicin fluorescence (gray) during daunorubicin injection into a mouse bearing ALL is shown. Time post injection is displayed. The second harmonic generation image of the collagen in the bone (blue) is displayed to show bone marrow compartments within the calvarium. Matching time-lapsed blood, tissue, and cellular compartment fluorescent intensity from this mouse is shown in Figure S3 I and K.

**Video E6:**

**Time-lapsed imaging of cellular clumping and intermittent BMV opening and closing.**

Time-lapsed imaging of 150kDa TRITC dextran fluorescence (gray) during dextran injection into a mouse bearing ALL is shown. Time post injection is displayed. The second harmonic generation image of the collagen in the bone (blue) and GFP+ ALL fluorescence (green) are displayed to show bone marrow compartments within the calvarium and local ALL burden respectively. Opening and closing of vessels and cell clumping are illustrated in the video.
