# Supplementary figures and images for: Biophysical Characterization of the Leukemic Bone Marrow Vasculature Reveals Benefits of Neoadjuvant Low-Dose Radiation Therapy
Source: Int J Radiat Oncol Biol Phys. Author manuscript; Available in PMC 2021 Jan 1. (PMC7736317; doi:10.1016/j.ijrobp.2020.08.037)

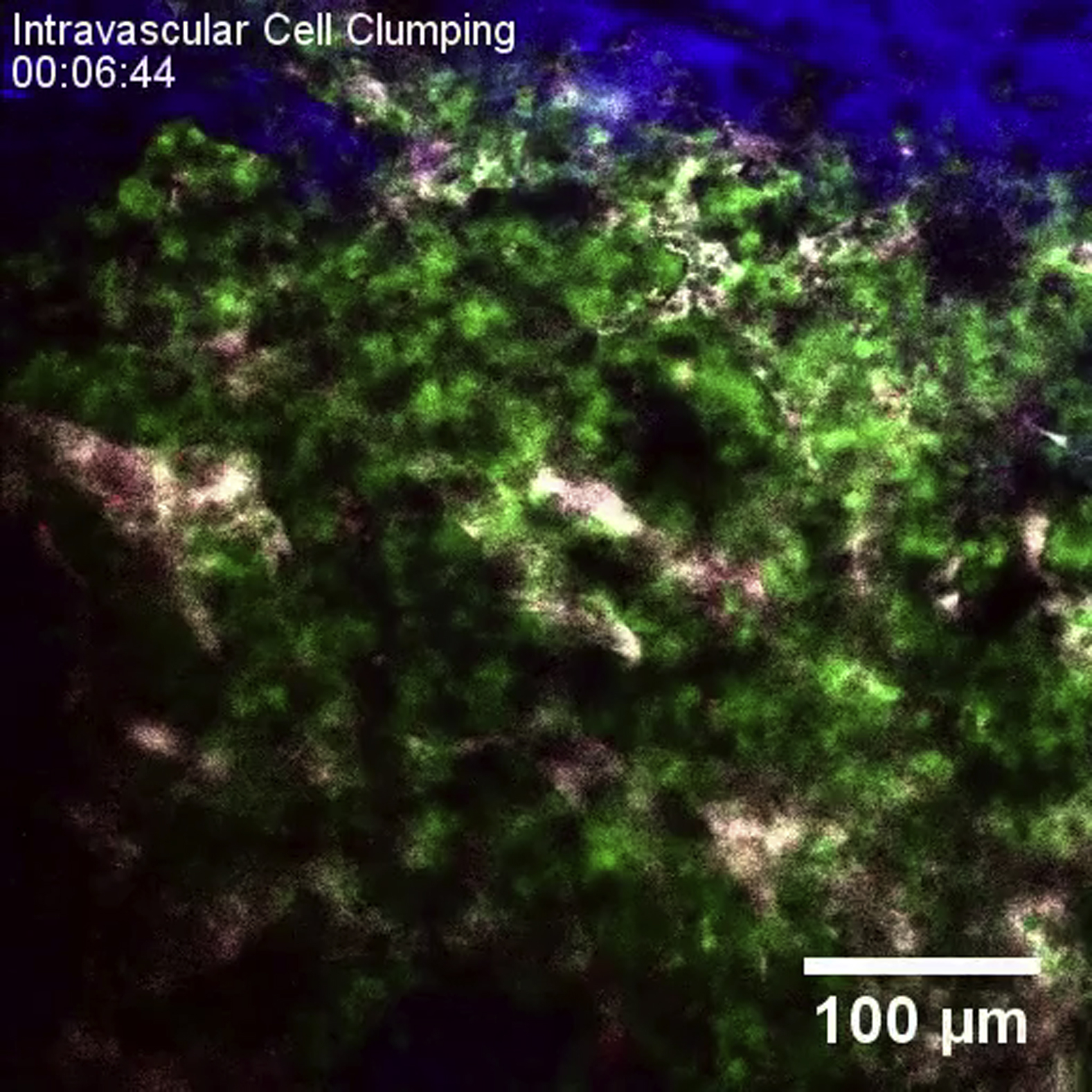

Supplement: mmc6fig [file NIHMS1623682-supplement-mmc6fig.jpg]

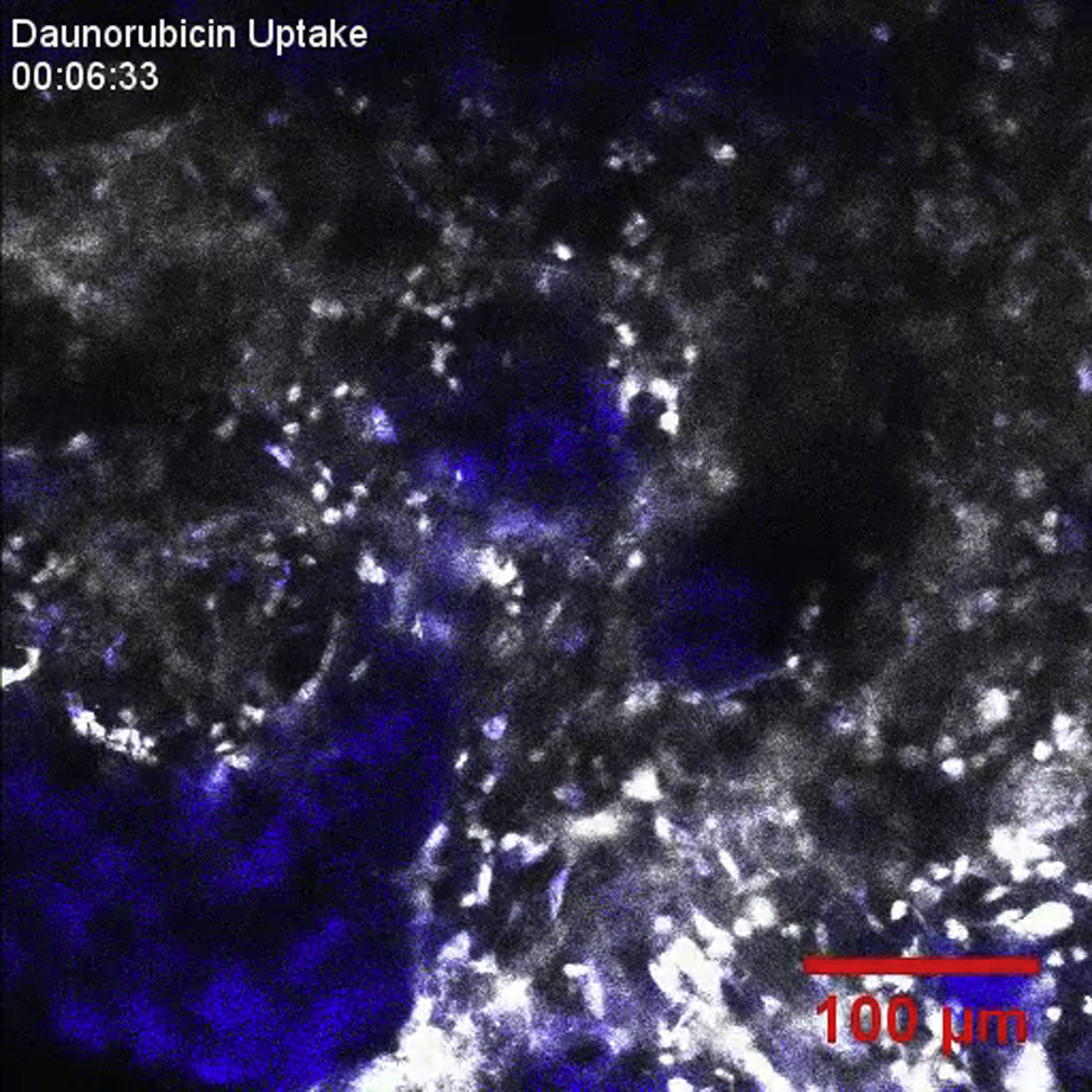

Supplement: mmc5fig [file NIHMS1623682-supplement-mmc5fig.jpg]

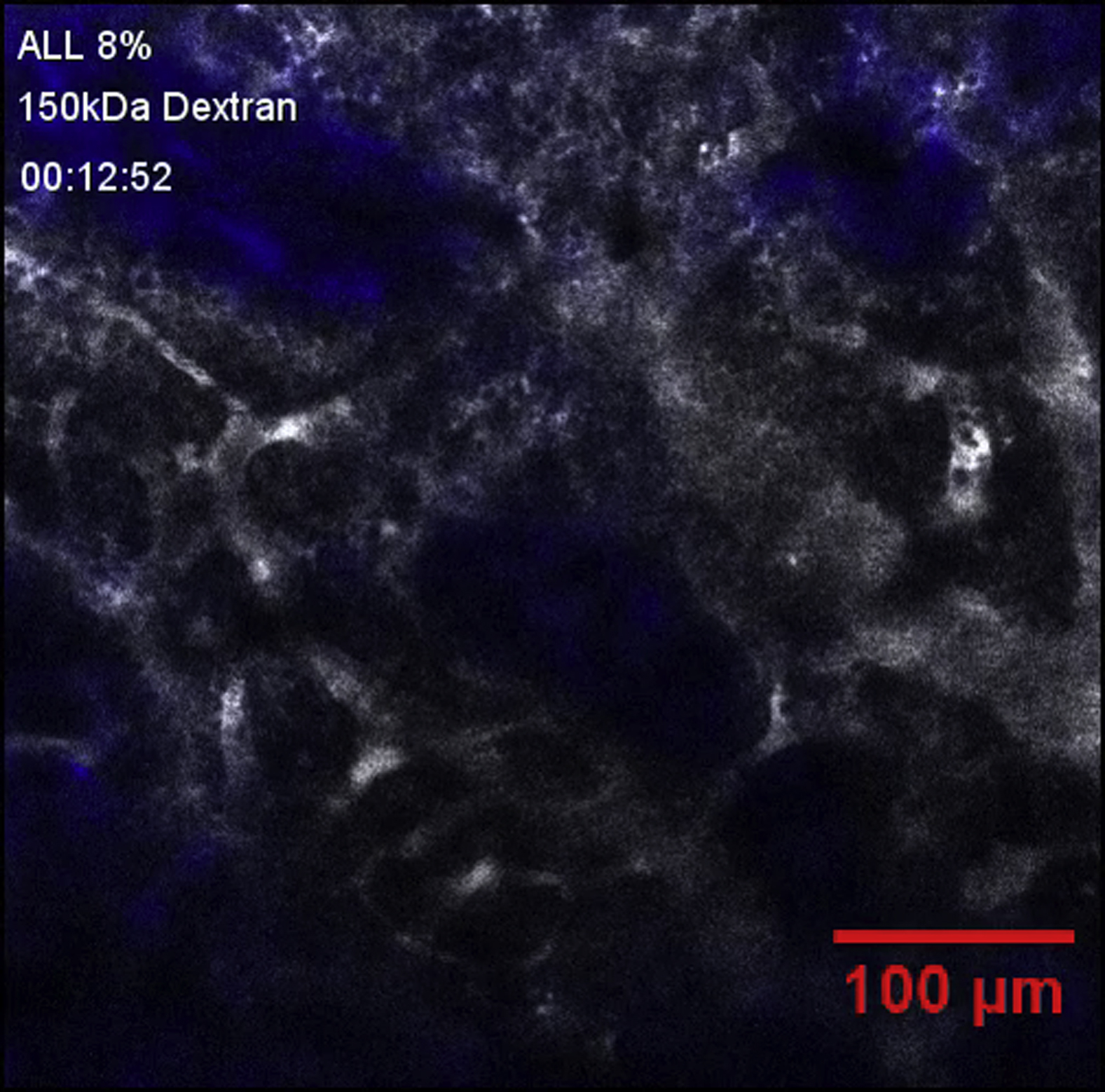

Supplement: mmc4fig [file NIHMS1623682-supplement-mmc4fig.jpg]

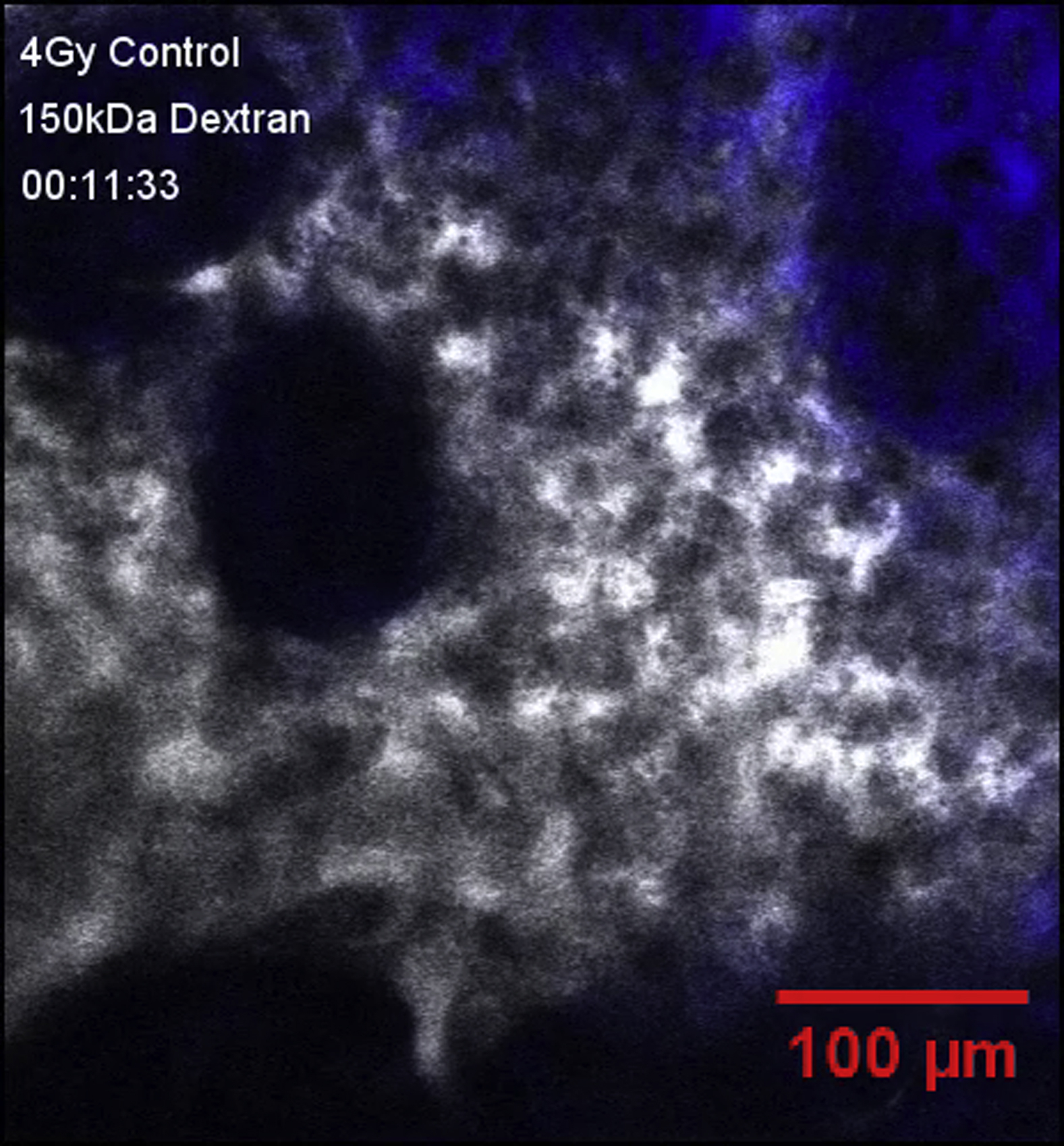

Supplement: mmc3fig [file NIHMS1623682-supplement-mmc3fig.jpg]

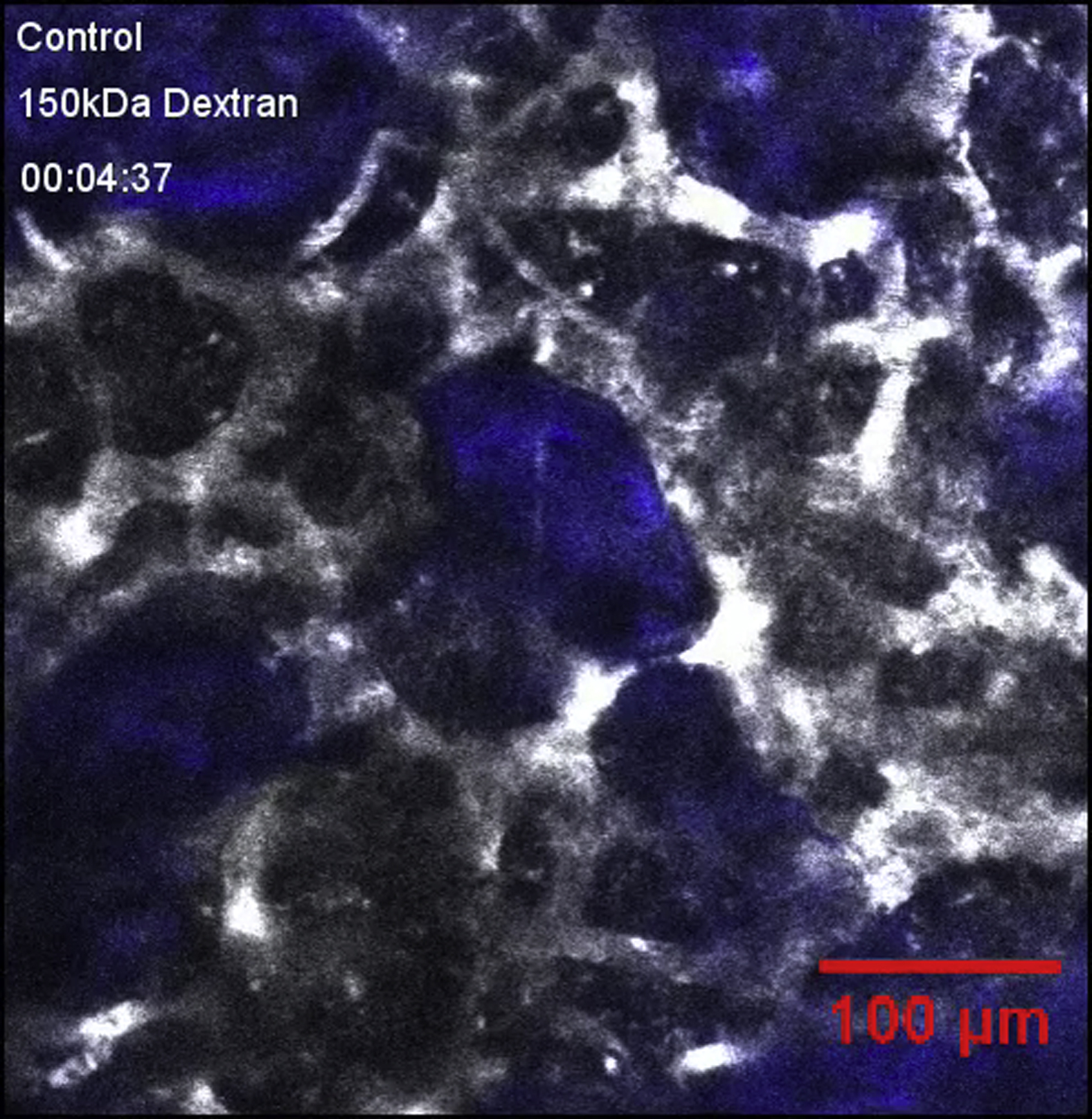

Supplement: mmc2fig [file NIHMS1623682-supplement-mmc2fig.jpg]

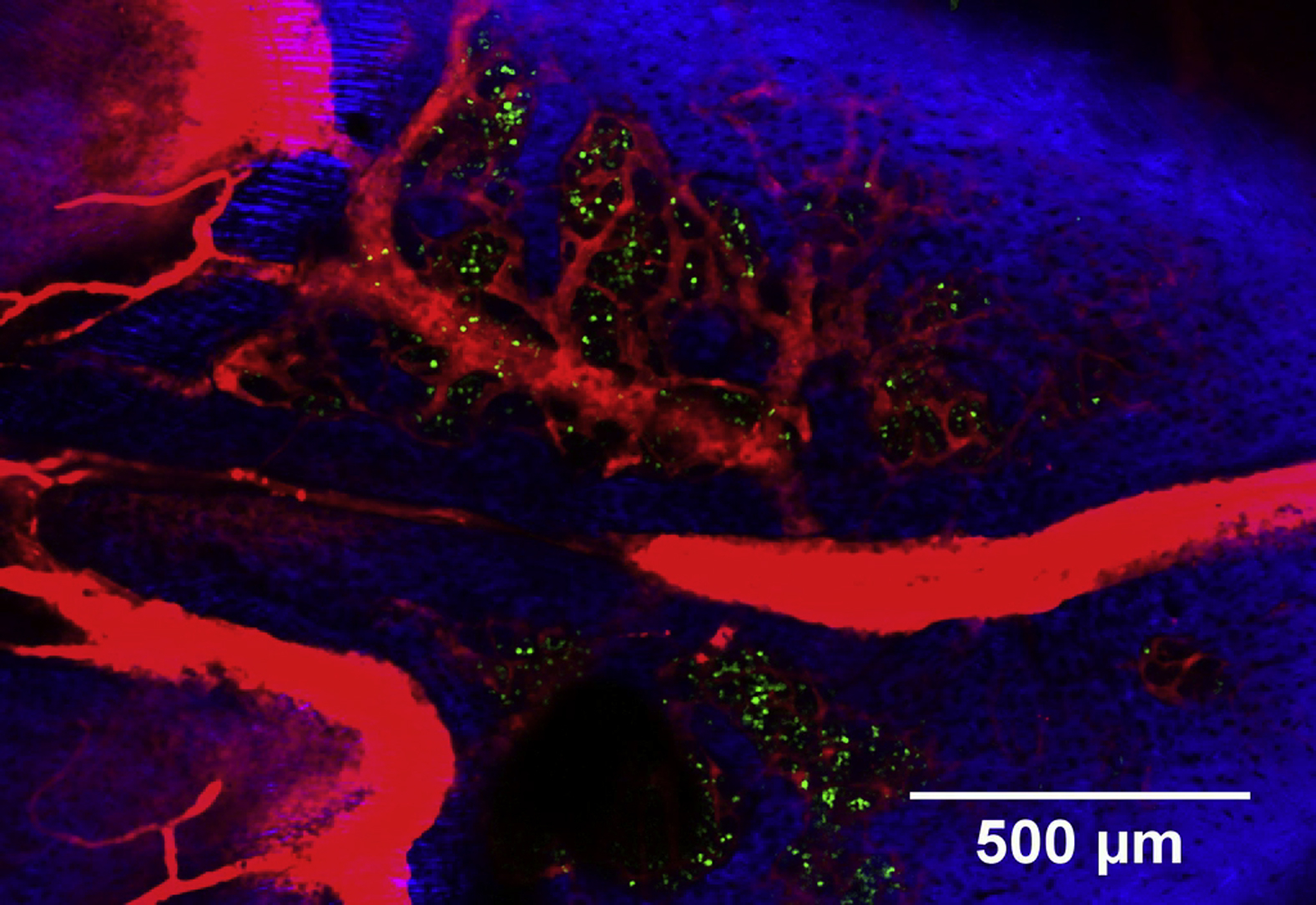

Supplement: mmc1fig [file NIHMS1623682-supplement-mmc1fig.jpg]
